# Structural and Functional Analyses of Human ChaC2 in Glutathione Metabolism

**DOI:** 10.3390/biom10010031

**Published:** 2019-12-24

**Authors:** Yen T. K. Nguyen, Joon Sung Park, Jun Young Jang, Kyung Rok Kim, Tam T. L. Vo, Kyu-Won Kim, Byung Woo Han

**Affiliations:** 1 Research Institute of Pharmaceutical Sciences, College of Pharmacy, Seoul National University, 1 Gwanak-ro, Gwanak-gu, Seoul 08826, Korea; kimyen@snu.ac.kr (Y.T.K.N.); wingpjs@snu.ac.kr (J.S.P.); nosvc4@snu.ac.kr (J.Y.J.); krkim85@snu.ac.kr (K.R.K.); qwonkim@snu.ac.kr (K.-W.K.); 2 Department of Biochemistry, Keimyung University School of Medicine, Daegu 42601, Korea; volutam@gmail.com

**Keywords:** breast cancer, ChaC2, enzyme, γ-glutamylcyclotransferase, GSH degradation, X-ray structure, redox homeostasis, flexible loop

## Abstract

Glutathione (GSH) degradation plays an essential role in GSH homeostasis, which regulates cell survival, especially in cancer cells. Among human GSH degradation enzymes, the ChaC2 enzyme acts on GSH to form 5-l-oxoproline and Cys-Gly specifically in the cytosol. Here, we report the crystal structures of ChaC2 in two different conformations and compare the structural features with other known γ-glutamylcyclotransferase enzymes. The unique flexible loop of ChaC2 seems to function as a gate to achieve specificity for GSH binding and regulate the constant GSH degradation rate. Structural and biochemical analyses of ChaC2 revealed that Glu74 and Glu83 play crucial roles in directing the conformation of the enzyme and in modulating the enzyme activity. Based on a docking study of GSH to ChaC2 and binding assays, we propose a substrate-binding mode and catalytic mechanism. We also found that overexpression of ChaC2, but not mutants that inhibit activity of ChaC2, significantly promoted breast cancer cell proliferation, suggesting that the GSH degradation by ChaC2 affects the growth of breast cancer cells. Our structural and functional analyses of ChaC2 will contribute to the development of inhibitors for the ChaC family, which could effectively regulate the progression of GSH degradation-related cancers.

## 1. Introduction

GSH is a crucial tripeptide (γ-glutamyl-cysteinyl glycine) that participates in diverse cellular functions, including cellular detoxification, redox signaling [1], cell proliferation, and apoptosis [2]. Disturbances of GSH homeostasis have been observed in many pathophysiological contexts [3]. In particular, an aberrant GSH level is correlated with tumor initiation, progression, and chemotherapeutic resistance [4,5]. As such, it has drawn attention in relation to cancer metabolism biology and treatment [4].

GSH homeostasis is balanced by GSH biosynthesis, transport and efflux of GSH, GSH-acting enzymes, and GSH degradation [6]. Although the contribution of GSH biosynthesis has been well studied, the GSH degradation pathway, which involves the initial cleavage of GSH, remains poorly understood. Several enzymes responsible for GSH degradation have been increasingly studied [6]. The human γ-glutamyltranspeptidase enzyme family comprises the first reported enzymes responsible for the GSH degradation pathway in the extracellular or vacuolar environment [7], which are able to cleave various γ-glutamyl substrates, including GSH-S-conjugate, GSSG (oxidized GSH), and γ-glutamyl compounds [8,9]. Recently, two isoform enzymes, ChaC1 and ChaC2, belonging to the γ-glutamylcyclotransferase (GGCT) family have drawn attention due to their ability to cleave the γ-glutamyl group of glutathione, resulting in 5-l-oxoproline and cysteinyl-glycine (Cys-Gly), specifically in the cytosol [10,11,12,13,14].

In humans, the GGCT-fold enzyme family is composed of γ-glutamylcyclotransferase (GGCT), γ-glutamylaminecyclotransferase (GGACT), and glutathione-specific γ-glutamylcyclotransferase (ChaC1 and ChaC2). Despite their low sequence identities, GGCT-fold enzymes exhibit a similar structural fold for their catalysis, including a YGSL motif and a typical catalytic Glu residue, as well as unique features, especially in the vicinity of the active site, which specifies the substrate repertoire [11,14]. The GGCT targets on γ-glutamyl derivatives, such as GSH [11] and GGACT, catalyzes γ-glutamylamines [12]. Interestingly, ChaC enzymes specifically act on GSH [15].

To date, two cytosolic ChaC isoforms, ChaC1 and ChaC2, have been identified in most mammalian tissues [13]. ChaC1 and ChaC2 share a 60% sequence identity but their physiological expression and catalytic efficiency are quite different [13]. ChaC2 exhibits a 10- to 20-fold lower catalytic efficiency than ChaC1 [13] and is constitutively expressed and involved in the “housekeeping” GSH metabolism [10]. In contrast, ChaC1 is an inducible enzyme that is expressed transiently in response to specific stress conditions or in tumor cells [13,16,17]. Recently, it was reported that ChaC2 directly targets GSH and regulates ChaC1 in order to control the GSH metabolism sustainably [10]. ChaC2 is known to affect the expression of the antioxidant master regulator nuclear erythroid-2-like factor (Nrf2) and glutamate cysteine ligase in the ChaC1-independent pathways [10]. To gain insights into how the ChaC enzymes specifically work on GSH and mediate their functions, homology models of ChaC1 based on the GGCT structure [14,15] and the crystal structure of yeast ChaC2 have been elucidated [13]. However, the structural features for the substrate specificities of the ChaC enzymes have not yet been fully described.

ChaC1 is known as a novel prognostic marker in many types of cancer [16,18,19]. The overexpression of ChaC1 has been found to result in the depletion of GSH, as well as enhanced apoptosis in a human embryonic kidney (HEK293) cell line [19,20]. Interestingly, ChaC1-overexpressing Hs578T breast cancer and HOC-7 ovarian carcinoma cell lines exhibited increased migration and proliferation [18]. Recent studies on ChaC2 have also shown differential functions in various situations. For example, ChaC2 is downregulated in gastric and colorectal cancer, and the overexpression of ChaC2 has been shown to inhibit cell proliferation [21]. On the contrary, the downregulation of ChaC2 resulted in the decrease of cell proliferation and the induction of cell death in human embryonic stem cells, and ChaC2 knockdown decreased the teratoma size and enriched teratoma adipocytes [10]. In addition, ChaC2 is exclusively up-regulated in ovarian cancer cell lines (OV-90, OVCAR3, SKOV3, TOV-21, TOV-112, and TOV-155) treated with drugs including cisplatin, paclitaxel, and topotecan [22]. GSH degradation is a well-known factor in apoptosis, cancer execution, and chemotherapeutic resistance in many cancer cells [16,23]; however, the correlation between ChaC family functions, GSH depletion, and breast cancer cell proliferation remains poorly understood [18].

Here, we report the crystal structures of ChaC2 with a unique flexible loop mediating a close contact with a crystallographically adjacent ChaC2 molecule, and ChaC2 E74Q/E83Q active site mutants with closed conformation. To further investigate the structure–function relationships, we conducted enzymatic assays, cell proliferation tests, multiple structural alignments, and molecular docking simulations. We identified the catalytic residues of ChaC2 responsible for the GSH degradation activity and propose that the long flexible loop regulates GSH degradation function. We associate the GSH degradation pathway mediated by ChaC2 with breast cancer proliferation and suggest a GSH degradation reaction mechanism. Taken together, our structural and biochemical analysis provide an insight into the cytosolic GSH degradation pathway. Our findings provide a basis for the design of novel inhibitors of ChaC family proteins in breast cancer, which often has issues of drug resistance.

## 2. Materials and Methods

### 2.1. Bioinformatics Analysis

The amino acid sequences of ChaC1, ChaC2, GGCT, and GGACT genes were obtained from UniProt [24]. Sequence alignments were performed using *Clustal Omega* [25] and visualized with *ESPRIPT3* [26]. The residues for substituted methionine were selected based on the secondary structure combined with solvent accessibility (SA) prediction, and the conserved sequence information of ChaC2 from various organisms [27]. The secondary structure and SA were predicted using the *PSIPRED* [28] and the *SABLE* server [29], respectively.

ChaC2 gene expression in different tissues was analyzed and visualized using the Oncomine database [30] (https://www.oncomine.org). The mRNA expression of ChaC2 in clinical invasive ductal breast carcinoma specimens was compared with that in normal tissue using Student’s *t*-test to generate the *p*-value. The cut-off *p*-value and fold change were defined as 0.05 and 1.3, respectively. Turashvili Breast, Karnoub Breast, and TCGA Breast were the main sources of the data that was analyzed. The Kaplan–Meier method was used to analyze the survival rate as a function of time. The Kaplan–Meier plot was acquired from the online Kaplan–Meier plotter (Breast Cancer) [31] (https://kmplot.com). The survival differences were conducted using the log-rank test.

### 2.2. Cloning and Protein Production

The cDNAs for human ChaC1 and ChaC2 were obtained from the Korean Human Gene Bank. The ChaC1 and ChaC2 genes were amplified by polymerase chain reaction (PCR) and inserted into pET-21a and pET-15b vectors between the BamHI and XhoI restriction enzyme sites with a hexa-histidine tag at the *C*-terminus and *N*-terminus, respectively. The vectors were transformed into the *Escherichia coli* C41 (DE3) strain. The transformed cells were incubated at 37 °C in Luria–Bertani (LB) medium containing ampicillin until the OD_600_ reached 0.6. Then, 0.4 mM IPTG (1-thio-ß-d-galactopyranoside) was added. The cells were incubated for further 20 h at 18 °C and harvested by centrifugation (Supra R22, Hanil Scientific Inc., Gyeonggi-do, Korea) at 6000× *g* for 10 min. The harvested cells were lysed by sonication for 10 min at 60% amplitude (SONICS & Materials Inc., Newtown, CT, USA) in buffer containing 20 mM Tris-HCl (pH 7.5), 500 mM NaCl, 35 mM imidazole and 1 mM phenylmethylsulfonyl fluoride. The lysates were centrifuged at 30,000× *g* for 1 h at 4 °C. The resulting supernatants were filtered using a 0.45 μM syringe filter device (Sartorius, Göttingen, Germany) and loaded onto a 5-mL HiTrap Chelating HP column (GE Healthcare, Chicago, IL, USA) pre-charged with Ni^2+^ and equilibrated with buffer containing 20 mM Tris-HCl (pH 7.5), 500 mM NaCl, and 35 mM imidazole. After washing with the buffer used in equilibration, the retained proteins were eluted by the addition of an increasing gradient of buffer containing 20 mM Tris-HCl (pH 7.5), 500 mM NaCl, and 1 M imidazole. The eluted fractions were dialyzed with buffer containing 20 mM Tris-HCl (pH 7.2) and 30 mM NaCl before loading onto a 5 mL HiTrap Q HP column (GE Healthcare). The bound proteins were eluted by adding an increasing gradient of buffer containing 20 mM Tris-HCl (pH 7.2) and 1 M NaCl. The eluted fractions were pooled and loaded onto a HiLoad 16/600 Superdex 75 pg column (GE Healthcare) equilibrated with buffer containing 20 mM Tris-HCl (pH 7.1), 150 mM NaCl, 5 mM 1, 4-dithiothreitol (DTT), and 2% glycerol. The purities of the protein fractions were confirmed by SDS-PAGE.

The oligomeric state of the proteins was characterized by size-exclusion chromatography with HiLoad 16/600 Superdex 75 pg column (GE Healthcare) in the same buffer, at a constant flow of 1 mL/min. The column was calibrated under identical running conditions with molecular weight standard mixture (thyroglobulin 670 kDa, γ-globulin 158 kDa, ovalbumin 44 kDa, myoglobin 17 kDa, and vitamin B_12_ 1.35 kDa).

For selenomethionine (SeMet) incorporation, a ChaC2 L21M, L118M, and L181M mutant was overexpressed in *Escherichia coli* B834(DE3) cells. The ChaC2-mutant transformed cells were grown in M9 minimal medium supplemented with l-selenomethionine and other amino acids. Protein preparation steps were the same with native proteins. A protease cocktail (Calbiochem, San Diego, CA, USA) and 10 mM 2-mercaptoethanol were supplemented in the buffer used in cell lysis. Finally, 5 mM DTT was added to all the purification buffers. 

### 2.3. Mutagenesis

ChaC2 mutants were generated using the QuickChange II Site-Directed Mutagenesis Kit (Agilent Technologies, Santa Clara, CA, USA). The recombinant pET-15b vector with ChaC2 gene was used as a template for mutagenesis. The mutant vectors were sequenced to confirm the presence of the desired mutation.

### 2.4. Crystallization

The crystallization conditions for ChaC2 were screened using commercial screening kits (Hampton Research, Aliso Viejo, CA, USA). The ChaC2 crystals were grown by using the vapor diffusion hanging drop method with a reservoir solution containing 14% (*w*/*v*) polyethylene-glycol 8000 (PEG 8000), 14% ethylene glycol, and 100 mM HEPES (pH 7.5) at 22 °C. The crystals of ChaC2 L21M, L118M, and L181M mutant and its SeMet-derivative were grown using a modified crystallization condition containing 14% (*w*/*v*) PEG 8000 and 100 mM Tris (pH 8.0) supplemented with 5% (*w*/*v*) PEG 200 for the ChaC2 mutant, and 2% pentaerythritol ethoxylate (3/4 EO/OH) for the SeMet-derivative, respectively. The crystals of ChaC2 E83Q and E74Q mutants were grown with 3 M sodium acetate trihydrate (pH 7.0). The crystals were improved with the addition of 10 mM GSH or 5-l-oxoproline to the crystallization solution.

### 2.5. X-ray Diffraction Data Collection, Structure Determination, and Refinement

Before the collection of the X-ray diffraction data, the ChaC2 crystals were cryoprotected in reservoir solution supplemented with 26% ethylene glycol (for the ChaC2 of ChaC2 L21M, L118M, and L181M mutants and SeMet-labeled crystals) or 20% glycerol (for ChaC2 E83Q and ChaC2 E74Q crystals) before flash freezing in a nitrogen-gas stream at 100 K. The X-ray diffraction data were collected on the beamlines 5C and 7A at the Pohang Accelerator Laboratory (Pohang, Korea) or Spring-8 (Osaka, Japan). The collected data were indexed, merged, and scaled using the *HKL2000* package [32]. The initial model of the SeMet-derived crystal of ChaC2 L21M, L118M, and L181M mutants was obtained by the wavelength anomalous dispersion (MAD) method using the *Autosol* program in *Phenix* software (Phenix 1.15.2, Berkeley, CA, USA) package [33]. The protein structure model was constructed using the *Autobuild* routine in the *Phenix* software package. The initial model from the MAD method was used as a template for molecular replacement using the *Phaser* program [34]. The ChaC2 model without the loop2 region was used as a temple for the ChaC2 E74Q structure determination by molecular replacement (MR) method. The ChaC2 E74Q model was used as template for the ChaC2 E83Q structure determination. Further iterative manual building and the refinement of models were carried out using *Coot* [35] and *CCP4i* [36]. The quality of the overall geometry and conformation were validated using the *MolProbity* program [37]. Before Protein Data Bank (PDB) deposition, *PDB-REDO* [38] was performed to complete the structures.

### 2.6. Cell Culture and Preparation of ChaC2-Overexpressing Cells

HEK293 and MCF-7 breast cancer cell lines were obtained from American Type Culture Collection (Manassas, VA, USA). The cells were cultured in Dulbecco’s modified Eagle’s medium (HyClone, Hudson, NH, USA) supplemented with 10% fetal bovine serum and 1% penicillin/streptomycin (Gibco, Waltham, MA, USA) in a humidified atmosphere of 5% CO_2_ at 37 °C. The ChaC2, ChaC2 E74Q, and ChaC2 E83Q were cloned into pEGFP-C3 vectors using the KpnI and BamHI restriction enzymes. The recombinant plasmids were transfected into the cells using polyethylenimine reagent (Sigma Aldrich, St. Louis, MO, USA) according to the manufacturers’ protocol at a rate of 2:1 (μL of polyethylenimine/g of plasmid DNA). The transfection efficiency was examined using a fluorescent microscope (Niko, Tokyo, Japan). To verify the successful overexpression of ChaC2 in these cell lines, western blotting was performed with the transfected cell lysates. The exogenous protein expression levels of GFP-ChaC2 were compared to those of an empty pEGFP-C3 vector (Mock).

### 2.7. Quantification of GSH

The Mock, ChaC2, ChaC2 E74Q, and ChaC2 E83Q overexpressed HEK293 cells were harvested and lysed as described in a previous study [39]. Briefly, 1 × 10^6^ cells were suspended in 25 μL of assay buffer with 175 μL of 5% meta-phosphoric acid before sonicating for 2 min with vortexing every 30 s. The sonicated cells were centrifuged at 12,000× *g* for 10 min at 4 °C before the assay. The supernatant samples were then diluted 50-fold and the concentration of GSH was immediately measured using the OxiSelect Total Glutathione Assay Kit (Cell Biolabs, Inc., San Diego, CA, USA). First, 50 μL of the buffer, standards, and samples were transferred to 96-well plates. Then, 50 μL of 5, 5′-dithiobis (2-nitrobenzoic acid) solution, 50 μL of reductase, and 50 μL of nicotinamide adenine dinucleotide phosphate were serially added. Immediately after, the absorbance of the samples at 412 nm was measured every 30 s for 2 min using a microplate reader (Spectra MAX M5; Molecular Devices, Sunnyvale, CA, USA). The GSH concentration was calculated from the absorbance. All measurements were conducted in triplicate.

For the in vitro enzymatic assay, 0.1 g/L of purified ChaC1, ChaC2, ChaC2 E74Q, and ChaC2 E83Q were incubated with 5 mM GSH in 100 μL at 37 °C for 1 h. Then, the reactants were heat-inactivated at 95 °C for 5 min. The samples were diluted 80 times before measuring the GSH concentration.

### 2.8. Viability Assay (MTT Assay)

The Mock, ChaC2, ChaC2 E74Q, and ChaC2 E83Q overexpressed breast cancer cells were seeded onto 96-well plates at a density of 1 × 10^4^ cells/well. After 48 h, 10 μL of the EZ-CYTOX solution from the Cell Viability, Proliferation & Cytotoxicity Assay Kit (DoGen, Seoul, Korea) was added to 100 μL of cell culture per well and incubated for 4 h for color development. The absorbance was measured at 450 nm to determine the number of viable cells. Each assay was performed at least three times.

### 2.9. Colony-Forming Assay

The transfected cells were seeded at a density of 1000 cells/well on 6-well plates and allowed to grow for 2 weeks. The colonies were fixed with 100% methanol, stained with 0.005% crystal violet, and counted. The experiments were repeated three times.

### 2.10. Immunoblotting

The cellular proteins were extracted using cell lysis buffer containing 20 mM Tris-HCl (pH 7.5), 150 mM NaCl, 0.1 mM ethylenediaminetetraacetic acid, 0.1% Triton X-100, and protease inhibitor cocktail (Calbiochem). The total protein (20 μg) was separated via SDS-PAGE and electroblotted onto a PVDF membrane. The membrane was incubated overnight with the corresponding primary antibodies, followed by the secondary antibody conjugated to horseradish peroxidase at 4 °C. Visualization was performed using ECL plus (GE Healthcare) and LAS-4000 (GE Healthcare). Anti-GFP (B-2, sc-9996) and anti-β-actin (I-19, sc-1616) antibodies were purchased from Santa Cruz Biotechnology, Dallas, TX, USA.

### 2.11. Docking Study

The crystal structures of ChaC2 E74Q and GGACT were used to calculate the binding energies of the GSH and 5-l-oxoproline to the protein. The calculation was implemented using *AutoDock Vina* software (AutoDock Vina 1.1.2, La Jolla, CA, USA) [40] with a grid map defined in 40 × 40 × 40 Å^3^ dimensions to contain the proposed active sites. The conformations of the ligands and receptors were fixed. The program was run in score mode with an exhaustiveness value of 20.

### 2.12. Surface Plasmon Resonance Experiment

Surface plasmon resonance (SPR) binding assays were performed using a carboxymethyl dextran (CM5) sensor chip on a Biacore T200 instrument (GE Healthcare). The amine coupling for ligand immobilization was performed at a flow rate of 5 μL/min. The chip was activated with a mixture of *N*-hydroxysuccinimide and *N*-Ethyl-*N′*-(dimethylaminopropyl) carbodiimide hydrochloride at a ratio of 1:1 for 400 s. Then, 13.3 μg/mL of ChaC2, ChaC2 E74Q, ChaC2 E83Q, and ChaC2 E74Q E83Q were diluted in 10 mM sodium acetate (pH 5.5) and injected until the immobilization level reached at 3000 RU. The remaining activated carboxyl groups were deactivated with 1 M ethanolamine at pH 8.5 for 400 s. The control experiment in reference flow cells was treated identically with BSA (bovine serum albumin) protein. The multi-cycle analysis was performed at a flow rate of 30 μL/min. GSH at concentrations of 1.95, 3.91, 7.81, 15.63, 31.25, and 62.50 μM in running buffer (150 mM NaCl, 10 mM HEPES-NaOH pH 7.2, 0.005% p20) was injected over the chip for 240 s, followed by dissociation for 600 s in a separate analysis cycle. The sensor chip surface was regenerated with 5 mM NaOH between cycles. Data were fitted using the simple bimolecular 1:1 Langmuir isotherm binding model. The equilibrium dissociation constant (*K_D_*) was determined using Biacore T200 evaluation software 3.0 (GE Healthcare).

### 2.13. Statistical Analysis

All statistical results are presented with the mean ± standard error of the mean (SEM) (error bars) of three independent experiments. The *p*-values were assessed using the two-tailed Student’s *t*-test. A *p*-value below 0.05 was considered as significantly different (*** *p* < 0.005; ** *p* < 0.01; * *p* < 0.05; n.s. (no significance), *p* ≥ 0.05).

### 2.14. Accession Numbers

The coordinates and structure factors for wild type ChaC2, ChaC2 E74Q, and ChaC2 E83Q have been deposited in the PDB with accession IDs, 6K95, 6KY0, and 6KY1, respectively.

## 3. Results

### 3.1. The ChaC2 Structure was Determined Through Rational Met-Substitution for MAD Phasing

ChaC2 does not share a reasonably high sequence similarity to proteins with known structures, which hindered the MR method for phasing. The subsequent multiple isomorphous replacement (MIR) method also failed to solve the phasing problem of the ChaC2 diffraction data set. In addition, ChaC2 contains only one Met residue at the *N*-terminus and the first Met was not sufficient to solve the phasing problem by MAD using the SeMet-derivatized ChaC2 protein with 184 amino acid residues. To overcome this lack of anomalous signaling, the Met-substitution method was used [29,41]. To this end, we initially chose the Leu and Ile residues located at the junction of two different secondary structure regions, which normally exhibited average or high predicted-solvent accessibility. Therefore, we used the *SABLE* server [29], which predicted the solvent accessibility bases on the secondary structure of the protein and suggested a number of potential residues to be replaced with Met (Figure S1). In order to further optimize the Met-substitution residues, we intensively investigated the amino acid sequence conservation among homologous ChaC2 proteins. Taken all together, we selected five residues: Leu21, Leu52, Leu119, Ile152, and Leu181, for Met-substitution (Figure S1). We attempted various combinations of Met-substitution with the five residues to produce single crystals for the structure determination of ChaC2. Consequently, we found that the ChaC2 mutant with L21M, L119M, and L181M yielded good diffracting-quality crystals, which allowed us to solve the crystal structure of ChaC2 at 2.8 Å resolution, using the MAD method. The data collection and structure refinement statistics are summarized in Table 1. 

**Figure 1:**
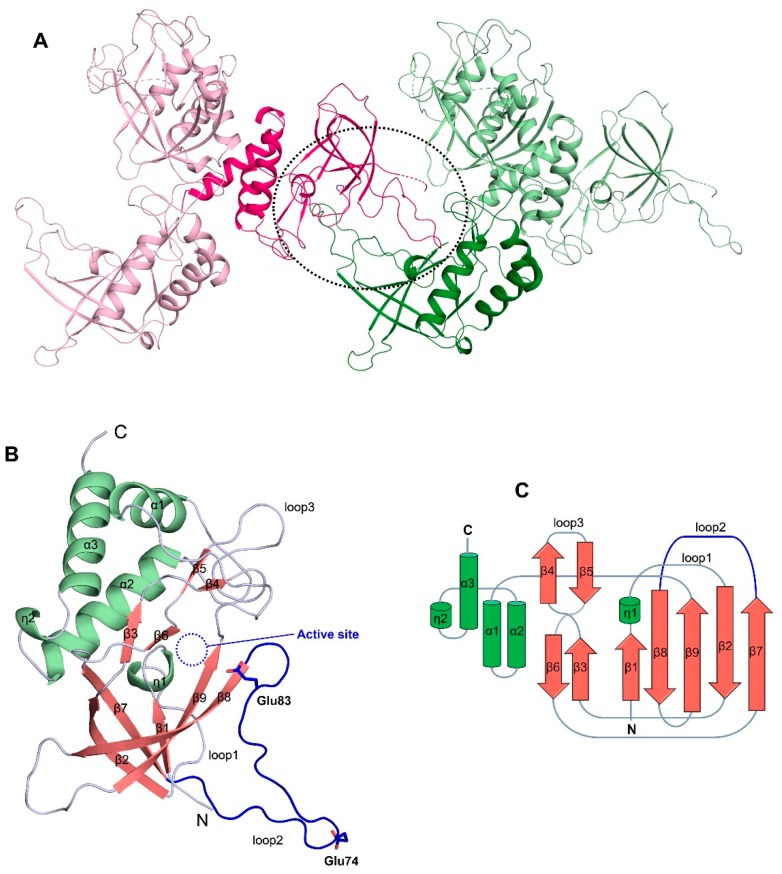
Overall structure of ChaC2. (**A**) Schematic representation of ChaC2 molecules in the crystal. ChaC2 molecules of the crystallographic dimer from two nearby an asymmetric unit (ASU) are colored in pink and green, respectively. The crystallographic dimer formed by flexible loop2 is denoted with a black dotted circle. (**B**) Structure of ChaC2. Helices, β-strands, and loops are colored in green, salmon, and light blue, respectively. The active site of ChaC2 is donated with a blue dotted circle. Loop2 is colored in blue. The side chains of Glu74 and Glu83 are shown as stick models. (**C**) Topology diagram of ChaC2. The secondary structure elements are denoted as in (**B**).

After several rounds of iterative model building and refinement of the SeMet-derivatized ChaC2 structure, unexpected electron densities were found adjacent to the suggested active site cavity. Furthermore, the electron maps of the Val60–Asp80 region could not be well defined, despite the overall electron map quality of ChaC2 being clear. With the native ChaC2 crystal structure at a higher resolution (2.3 Å), we were able to finally determine that these electron densities came from the loop region (residues Val60–Asp80) of the adjacent ChaC2 molecule. There were three ChaC2 molecules in an asymmetric unit (ASU) (Figure 1A). Each ChaC2 molecule formed a crystallographic dimer with an adjacent ChaC2 molecule (Figure 1A). The *R_work_* and *R_free_* values for the ChaC2 structure were 0.24 and 0.27, respectively. The data collection and refinement statistics are summarized in Table 2.

### 3.2. The Overall Structure of ChaC2 Adopts the GGCT Fold

The ChaC2 structure exhibited a mixed α/β topology with nine β-strands (β1–β9), three α-helices (α1–α3), and two 3_10_-helices (η1 and η2) (Figure 1B,C). The five β-strands, β1, β2, β7, β8, and β9, form a central antiparallel β-sheet (β1↑β2↓β7↑β8↓β9↑). The connecting loop between β-strand β3 and β4 crosses over and twists around the corresponding loop between β-strand β6 and β5 (Figure 1B). In addition, the ChaC2 contains the conserved catalytic cavity that is observed among proteins containing the GGCT fold [11], and formed by the key ^6^YGS^8^ motif in loop1 (residues Gly5–Arg30), Tyr87 and Tyr109 in β8 and β9, β1, Tyr144 in helix α2, and hydrophilic residues Asn34, His39, and Arg40 in loop3 (residues Asn34–Gly46) (Figure 1B and Figure S1). As mentioned earlier, the ChaC2 structure exhibited a unique flexible conformation with loop2 (residues Val60–Asp80), flipping out from the core domain (Figure 1B). Thus, the Glu residues, which are generally known as general acid/base catalytic residues in the GSH degradation reaction, were ambiguous in the activity cavity of one ChaC2 monomer (Figure 1B).

### 3.3. Flexible Loop2 in the Crystallographic ChaC2 Dimer Represents an Open Conformation

**Figure 2:**
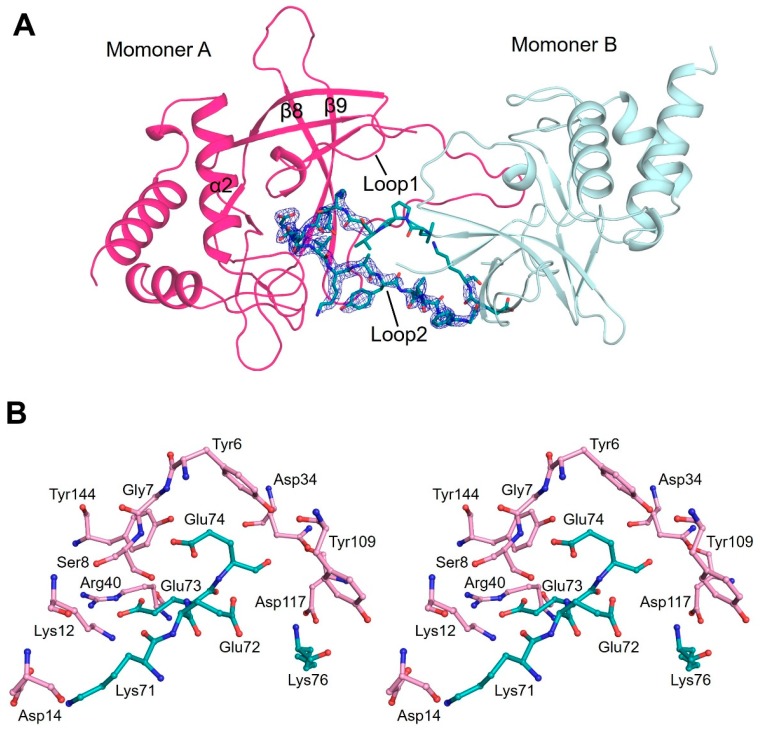
Open conformation of ChaC2. (**A**) Crystallographic ChaC2 dimer with long flexible loop2. Two ChaC2 monomers are colored in pink and pale cyan, respectively. The loop2 residues are represented as green stick models and their 2*mFo*-*DFc* electron densities of *2.0σ* are shown with blue meshes. (**B**) A stereo-view of the dimeric interface of ChaC2. The residues from the two interacting ChaC2 molecules are denoted with pink and cyan models, respectively. The nitrogen and oxygen atoms are denoted in blue and red, respectively.

In each ChaC2 molecule in ASU, the long flexible loop2 protruded from the center crystallographic trimer and formed an unprecedented dimer with the adjacent ChaC2 molecule, which we call an open conformation (Figure 2A). When we calculated the 2*mFo*-*DFc* map at 2σ, the loop2 region exhibited unambiguous positive electron densities that confirmed the long flexible loop in a crystallographic ChaC2 dimer (Figure 2A and Figure S2). Analysis of the oligomeric state of the ChaC2 structures using *PISA* web server [42] also indicated the formation of a stable homodimer with a relatively large buried area of 1349.4 Å^2^, covering 13.1% of the ChaC2 monomer surface area. The dimer interface included the loop2 region from one ChaC2 monomer and the loop1 from the other ChaC2 monomer (Figure 2A). Coincidently, the crystallographic dimer interface was located adjacent to the proposed active site cavity, which is characterized by loop1, α2, β8, and β9, as previously mentioned. In addition, the crystallographic ChaC2 dimer with long flexible loop2 established stable interactions via numerous hydrogen bonds and salt bridge networks in the predicted active site cavity (Table S1). Furthermore, Lys12 and Arg40 from one ChaC2 monomer formed salt bridges with the side chain of Glu73 from the other ChaC2 monomer. In addition, 22 hydrogen bonds were tightly formed between Ser8/Lys12/Arg40/Tyr109/Tyr144 from one ChaC2 monomer and Lys71/Glu73/Glu74/Lys76 from the other ChaC2 monomer (Table S1 and Figure 2B). Notably, among these residues, Ser8, Arg40, Glu74, Tyr109, and Tyr144 were strictly conserved among the GGCT enzymes and contributed to the active site cavity (Figures S1 and S4).

### 3.4. Single Mutations of E74Q and E83Q Induced Conformational Changes in the Flexible Loop2 Region of ChaC2 and Resulted in a Closed Conformation

**Figure 3:**
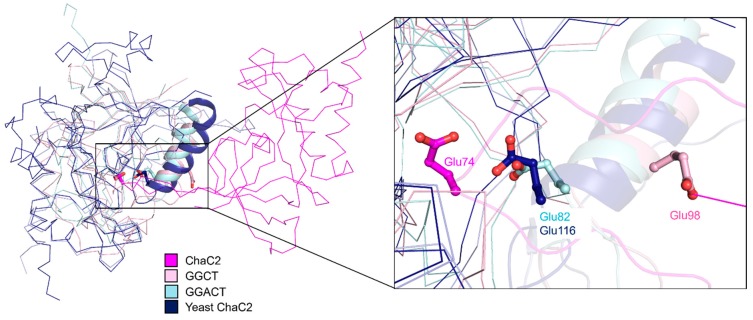
Structural superposition of the structures of human ChaC2, GGCT (PDB ID: 2PN7), GGACT (PDB ID: 3JUC), and yeast ChaC2 (PDB ID: 5HWI). Two human ChaC2 molecules in a crystallographic dimer are colored in magenta and light blue, respectively. Human GGCT, GGACT, and yeast ChaC2 structures are colored in light pink, pale cyan, and blue, respectively. The Glu residues in the active site cavities of human ChaC2, GGCT, and GGACT and yeast ChaC2 are shown as stick models.

Despite many attempts, we were unable to determine any complex structure of ChaC2 with the substrate GSH or the products 5- l-oxoproline and Cys-Gly in the GSH degradation reaction, which would have revealed the exact binding mode or snapshot of the catalytic mechanism. Nevertheless, structural similarity analyses implemented with the coordinates of ChaC2 using the *DALI* web server [43] demonstrated that the overall structure of ChaC2 shares a highly conserved scaffold with the other GGCT protein structures (Table S2). ChaC2 showed high structural similarities to yeast glutathione-specific gamma-glutamylcyclotransferase (PDB ID: 5HWI, z-score = 19.2, sequence identity = 38%), human GGCT (PDB ID: 2PN7, z-score = 12, sequence identity = 21%), and human GGACT (PDB ID: 3JUC, z-score = 7.2, sequence identity = 21%). When the structure of the crystallographic ChaC2 dimer with flexible loop2 was superimposed with the structurally similar enzymes, the Glu74 residue in loop2 of ChaC2 was found to be located at the dimer interface and structurally equi-positional to other active-site Glu residues in the structurally similar enzymes [11,12,13] (Figure 3). Thus, we hypothesized that Glu74 plays a critical role not only in the catalytic function but also in the dimerization in a crystal via flexible loop2.

**Figure 4:**
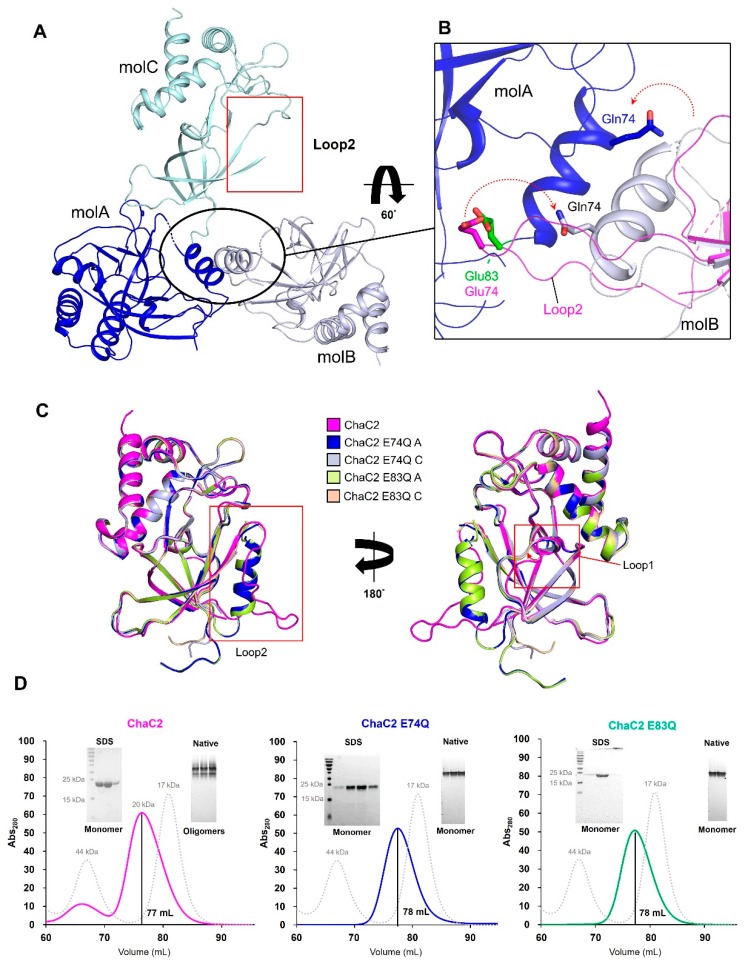
Conformation changes of ChaC2 E74Q and ChaC2 E83Q mutants. (**A**) Structures of the ChaC2 E74Q mutant. Three ChaC2 E74Q molecules (mol) in an ASU are denoted in blue (molA), white blue (molB), and pale cyan (molC), respectively. The loop2 region of molC is marked by a red circle. (**B**) Newly formed helices on the interface of ChaC2 E74Q molA and molB superimpose to the loop2 region of the ChaC2 monomer. Side chains of Gln74 and Glu83 of ChaC2 E74Q molA and molB are represented as stick models. The Glu83 of ChaC2 E74Q molA and Glu74 of ChaC2 monomer are colored in green and magenta, respectively. The Gln74 of ChaC2 E74Q molA and molB are colored in blue and white blue, respectively (**C**) Superimposition of ChaC2 and its mutants. The structure of ChaC2 (magenta), ChaC2 E74Q (blue and light blue), and ChaC2 E83Q (green and wheat) showing the different conformations of loop2 (left) and loop1 (right) are superimposed. Loop2 and loop1 are donated with red boxes. (**D**) Size-exclusion chromatograms for ChaC2 (pink line), ChaC2 E74Q (blue line), and ChaC2 E83Q (green line). ChaC2 (20 kDa), ChaC2 E74Q, ChaC2 E83Q, and calibrants were loaded onto a HiLoad Superdex 75 pg column at a flow rate of 1 mL/min. The eluted proteins were monitored at 280 nm. The chromatogram of the calibration mixture (ovalbumin 44 kDa and myoglobin 17 kDa) are shown in gray. The SDS-PAGE and native-PAGE gel of the main peak protein fractions are shown beside the chromatograms.

To validate our hypothesis, we generated the ChaC2 E74Q mutant and determined its crystal structure. The ChaC2 E74Q structure was solved at 2.2 Å resolution (Table 2), with one homo-trimer of ChaC2 E74Q A, B, and C in ASU (Figure 4A). Compared with the ChaC2 monomer in an open conformation, ChaC2 E74Q A (B) adopted a helix conformation in the equivalent position of the ChaC2 loop2, which is mainly involved in dimerization (Figure 4A,B). The helix withdrew inward towards the center of a monomer molecule and did not form the crystallographic ChaC2 dimer with the long flexible loop2, resulting in a closed conformation (Figure 4B). The Gln74 withdrew from the adjacent active site cavity then protruded outward towards the rear of the newly formed helix (Figure 4B). Interestingly, neither loop2 nor the helix conformation was visible in the equivalent region of ChaC2 E74Q C despite the overall fold of ChaC2 E74Q C being identical to that of the ChaC2 monomer (Figure 4A,C and Figure S3). This finding suggests that ChaC2 E74Q C is a snapshot of a transition from a loop conformation to a helix.

**Figure 5:**
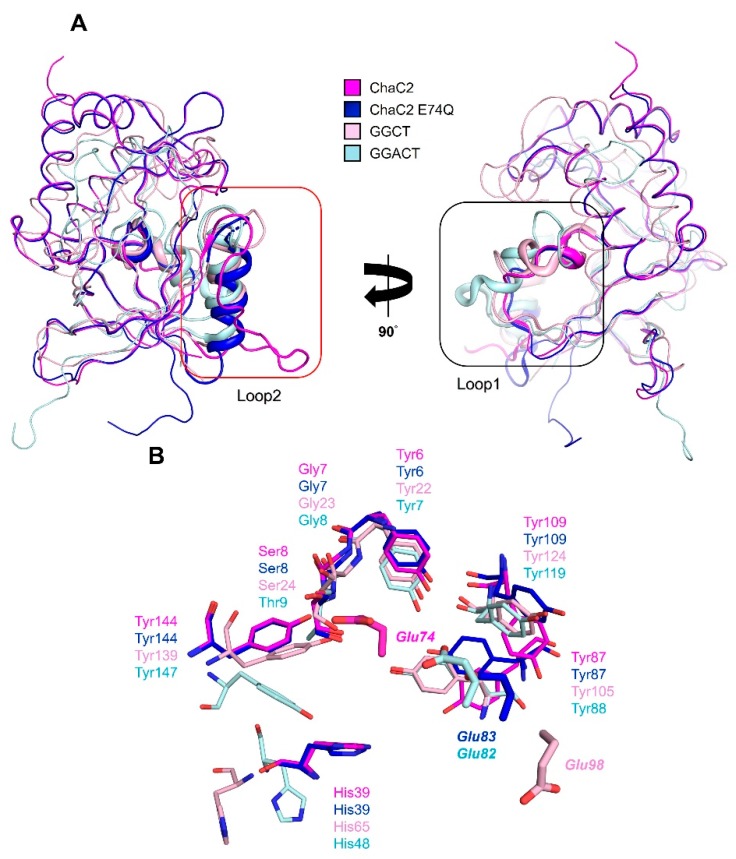
Structural comparison of human ChaC2, human GGCT, and human GGACT. (**A**) The structures of ChaC2 (magenta), ChaC2 E74Q (blue), GGCT (PDB ID: 2RBH, pink), and GGACT (PDB ID: 3JUC, cyan) are superimposed. The loop2 and loop1 regions of ChaC2 are indicated by red (left) and black (right) boxes, respectively. (**B**) The substrate-binding residues of GGCT and GGACT are denoted as pink and cyan stick models, respectively. ChaC2/ChaC2 E74Q residues overlapped with the substrate-binding residues of GGCT and GGACT are denoted as magnetic/blue stick models.

Surprisingly, the ChaC2 E74Q A structure was found to have Glu83 also move along with the newly formed helix, to become located at the active site cavity of ChaC2 E74Q A (B) (Figure 4B). The Glu83 replaced the Glu74 and extended into the active site cavity of the new helix conformation monomer (Figure 4B). As a result, Glu83 aligned very well with other catalytic Glu residues of GGCT enzymes and with Glu74 in the crystallographic ChaC2 dimer with flexible loop2 (Figure 4B and Figure 5B). Furthermore, our amino acid alignment showed that Glu83 was strictly conserved with other catalytic residues of yeast ChaC2, human GGCT, human GGACT, and human ChaC1 (Figure S4). Therefore, we decided to investigate the function of Glu83 in greater detail. We generated a ChaC2 E83Q mutant and determined its crystal structure. The ChaC2 E83Q structure was solved at 2.0 Å resolution. The overall fold of the ChaC2 E83Q molecules, A and C, resembled those of ChaC2 E74Q, A and C, respectively (Figure 4C). The loop1 regions in both ChaC2 E83Q and ChaC2 E74Q were slightly rotated compared with that of ChaC2 (Figure 4C, right panel). Noticeably, the electron density around the loop2 region of the ChaC2 E83Q C molecule was more clear than that of the ChaC2 E74Q C molecule (Figure S3), suggesting that there is an existing partial-changing conformation, wherein loop1 seems to be involved in the mobility of loop2 during conformational switching.

To validate the oligomeric state of ChaC2 in solution, we implemented size-exclusion chromatography with a HiLoad Superdex 75 pg column followed by SDS-PAGE and native-PAGE analyses. The elution profile of ChaC2 showed a major peak corresponding to the monomer status, and the eluted proteins generated a single monomer band in SDS-PAGE and native-PAGE gels (Figure 4D). Interestingly, the protein band of the ChaC2 sample from the major peak was also distributed in discrete bands above the major band in native-PAGE, implying that ChaC2 molecules exist in a higher oligomeric state (Figure 4D, left panel). In the cases of ChaC2 E74Q and ChaC2 E83Q, the elution profiles from size-exclusion chromatography also showed a monomer peak, and the mutants of ChaC2 exhibited a single monomer band in SDS-PAGE and native-PAGE gels (Figure 4D, middle and right panels, respectively). These data indicate that ChaC2 could interact together and adopt higher oligomeric states, whereas ChaC2 E74Q or ChaC2 E83Q may exist as monomers in solution, which further supports the structures of the crystallographic ChaC2 dimer with flexible loop2 and ChaC2 E74Q and ChaC2 E83Q monomers.

### 3.5. Structural Comparison of Human ChaC2 and other GGCT Enzymes Reveals Flexibility in the Active Site Region

Despite sharing the conserved β-barrel fold where the substrate γ-glutamyl moiety binds, each GGCT protein contains a different number of appended α-helices, β-strands, and loops around the β-barrel topology to recognize substrates specifically [11,12,13]. The l-γ-glutamyl moiety of GGCT links to l-α-amino acids, but that of GGACT links to extended alkylamines [20]. In order to elucidate the structure-based GSH specific activity information, we compared the crystal structure of ChaC2 with other GGCT structures. ChaC2 exhibited remarkable structural differences in the loop2 and loop1 regions that lie in the active site, compared with GGCT and GGACT (Figure 5A). The loop2 region in the ChaC2 structure was involved in the open conformation, while the loop2 regions of ChaC2 E74Q and other GGCT proteins adopted a helical conformation (Figure 5A). Consequently, the active site cavity of ChaC2 was relatively flexible, compared with other structurally homologous enzymes.

The GGCT proteins generally have an acid/base Glu in their active site; Glu98 (in human GGCT) [12], Glu82 (in human GGACT) [13], and Glu116 (yeast ChaC2) [11] (Figure 3). To identify the equivalent catalytic Glu residue of ChaC2, we carefully investigated the active site residues of ChaC2 with other GGCT enzymes (Figure 5B). The superimposition of their structures clearly revealed that Tyr6, Gly7, Ser8, His39, Tyr87, Tyr109, and Tyr144 of ChaC2 were strictly conserved with other GGCT enzymes (Figure 5B). Interestingly, the catalytic Glu was aligned very well with both the Glu74 and Glu83 of ChaC2 and ChaC2 E74Q, respectively. Thus, we propose that ChaC2 has two alterative acid/base Glu residues, corresponding to two state conformations.

### 3.6. ChaC2 E74Q and ChaC2 E83Q Mutations Significantly Reduced GSH-Degradation Activities in Cell and In Vitro

**Figure 6:**
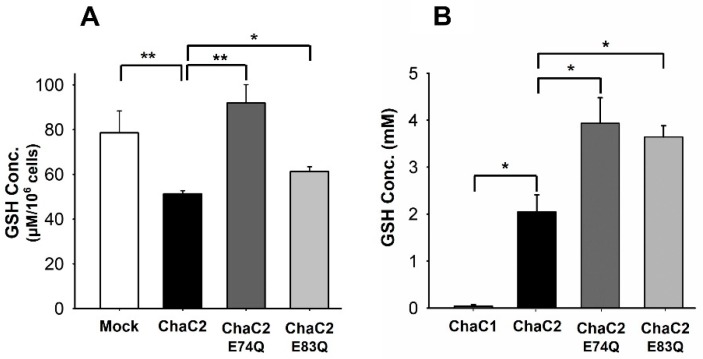
GSH level reduction in cells and in vitro by ChaC2. (**A**) The GSH levels in HEK293 cell lines transfected with Mock, ChaC2, ChaC2 E74Q, and ChaC2 E83Q-containing pEGFP-C3 plasmids were measured. (**B**) Briefly, 5 mM of GSH was incubated with ChaC1, ChaC2, ChaC2 E74Q, and ChaC2 E83Q and the levels of residual GSH were measured. Data represent the mean ± SEM (error bars) of three independent experiments. Asterisks indicate statistically significant differences; * *p* < 0.05; ** *p* < 0.01.

Recent studies have shown that the overexpression of ChaC2 decreases the GSH levels in yeast cells [13] and gastric cancer cells [21]. To further elucidate the functional importance of Glu74 and Glu83, we measured the GSH levels in ChaC2-, ChaC2 E74Q-, and ChaC2 E83Q-overexpressing HEK293 cell lines. ChaC2-overexpressing HEK293 cells showed lower GSH levels than Mock (Figure 6A), while ChaC2 E74Q- and ChaC2 E83Q-overexpressing HEK293 cell lines exhibited higher GSH levels than ChaC2-overexpressing cells (Figure 6A). The overexpression efficiency of exogenous ChaC2 proteins was verified by western blotting (Figure S5).

We also implemented in vitro enzymatic assays to compare the GSH degradation activity of ChaC2, ChaC2 E74Q, and ChaC2 E83Q with human ChaC1 as a positive control. After 1 h of reaction, ChaC1 degraded 99.14% of the total GSH; however, ChaC2 was unable to degrade GSH as efficiently as ChaC1. ChaC2 was only able to degrade 58.97% of the total GSH (Figure 6B), which is consistent with the previously reported results [10,13]. ChaC2 E74Q and ChaC2 E83Q exhibited lower GSH degradation activity than ChaC2, that is, 21.34% and 27.15% degradation of the total GSH, respectively (Figure 6B). In summary, our GSH degradation assay demonstrated that human ChaC2 significantly reduced the GSH levels in cells and in vitro, and that both Glu74 and Glu83 residues play important roles in GSH degradation mediated by ChaC2. In conclusion, ChaC2 has GGCT activity and both Glu74 and Glu83 residues are involved not only in the structural conformation but also in the activity of ChaC2 in cells and in vitro.

### 3.7. ChaC2 Overexpression Promotes Cell Proliferation in the MCF-7 Breast Cancer Cell Line

Recently, it has been reported that the downregulation of GGCT elicits an anti-proliferative effect in some cancer cell lines, such as breast cancer cell lines MCF-7, MCF-7/ADR, and MDA-MB-231 [44,45,46]. Interestingly, ChaC1 was suggested as a novel biomarker because the overexpression of mammalian ChaC1 and its related transcript variants has been previously found to promote cell proliferation in breast cancer cell lines Hs578T and BT-20 [18,47,48].

**Figure 7:**
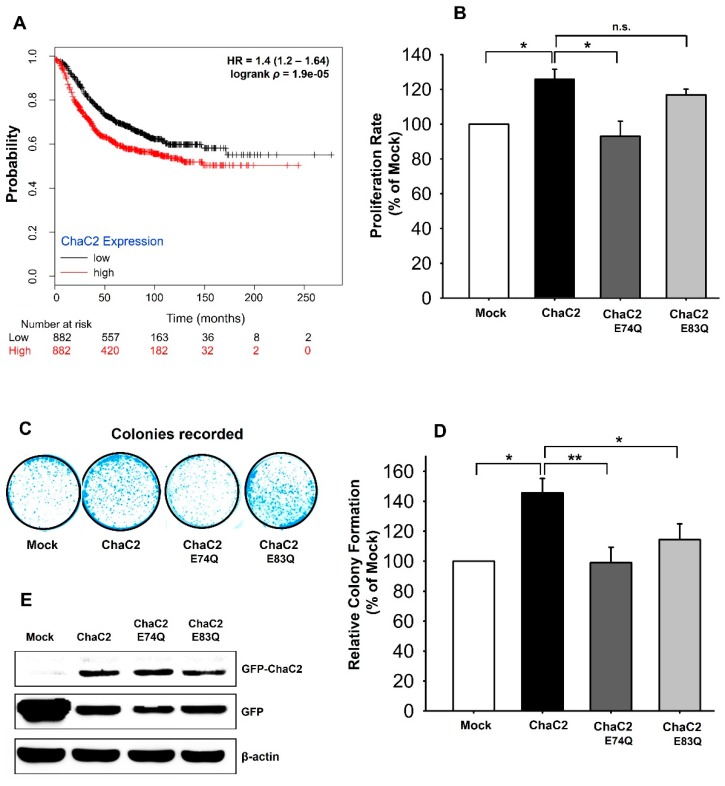
Proliferation of breast cancer cell lines by ChaC2 overexpression. (**A**) The Kaplan–Meier plots according to ChaC2 expression levels in patients with breast cancer. The plots show lower survival rates in patients with high ChaC2 expression compared to low ChaC2 expression. (**B**) Viability of ChaC2-overexpressed MCF-7 cells measured by MTT assay. The proliferation rate of cells is shown with relative percentages compared to the Mock-transfected cells. Data represent the mean ± SEM (error bars) of three independent experiments. Asterisks indicate statistically significant differences; * *p* < 0.05; ** *p* < 0.01; n.s. (no significance), *p* ≥ 0.05. (**C**,**D**) Colony forming assay with ChaC2-overexpressed MCF-7 cells. The representative photos were taken after 2 weeks from initial seeding (**C**). The relative percentages of the colony numbers of the ChaC2, ChaC2 E74Q, and ChaC2 E83Q-transfected cells to Mock-transfected cells are represented as the mean ± SEM (error bars) of three independent experiments (**D**). (**E**) The overexpression of ChaC2 in the ChaC2-transiently transfected MCF-7 cells. Cell lysates of the Mock, ChaC2, ChaC2 E74Q, and ChaC2 E83Q-transfected cells were analyzed by western blotting using a GFP antibody to detect GFP-tagged ChaC2 proteins.

When we analyzed the ChaC2 gene expression in breast cancer tissues from the online clinical database Oncomine, the TCGA, Turashvili Breast, and Karnoub Breast database showed that the transcriptional level of ChaC2 in invasive ductal breast carcinoma was significantly higher than in normal cells. In detail, the TCGA Breast source of ChaC2 (Reporter ID: A_23_P28571) reveals that when comparing 389 invasive ductal carcinomas to 61 breast samples, ChaC2 shows a 1.3 fold change in expression. The Turashvili Breast source of ChaC2 (Reporter ID: 235117_at) and Karnoub Breast source of ChaC2 (Reporter ID: 235117_at) also reveal that the ChaC2 gene expression fold changes were 4.5 and 1.8, respectively. In addition, the Kaplan–Meier plot of ChaC2 (Affymetric ID: 235117_at), according to gene overexpression in breast cancer patients and their overall survival information, showed that the survival rate of patients with breast cancer with high ChaC2 expression levels was significantly lower than that with low ChaC2 expression levels (*p* < 0.001) (Figure 7A). Notably, ChaC2 shares a high structural similarity with GGCT enzymes and a high amino acid sequence similarity with ChaC1. Based on these finding, we became interested in the biological effects of ChaC2 on breast cancer cell lines and related mechanisms. As such, we examined the effects of ChaC2 and ChaC2 active-site mutants (ChaC2 E74Q and ChaC2 E83Q) overexpressed in invasive ductal breast carcinoma MCF-7 cell lines on cell growth by MTT assays and colony-forming assays. At the same protein level overexpression, ChaC2 overexpression led to a significant increase in cell proliferation, compared to the Mock (Figure 7B). However, ChaC2 E74Q overexpression did not induce cell proliferation as in ChaC2 overexpression, while ChaC2 E83Q overexpression elicited a similar cell proliferation level as ChaC2 overexpression (Figure 7B).

To confirm the MTT assay results, we further implemented a colony-forming assay with ChaC2-overexpressing MCF-7 cell lines. After two weeks of transfection, we counted the ChaC2-overexpressing cell colony number and compared it with that of Mock treated-cells. Consistent with our MTT assay results, ChaC2-overexpressing cells formed more colonies than Mock treated-cells (Figure 7C,D). ChaC2 E74Q- and ChaC2 E83Q-overexpressing cells formed significantly less colonies than ChaC2-overexpressing cells (Figure 7D). The overexpression efficiency of the exogenous proteins after transiently transfecting ChaC2 and the Mock into MCF-7 cell lines was evaluated by western blotting (Figure 7E). The results showed a significant overexpression of ChaC2 in this breast cancer cell line and confirmed that the ChaC2 mutants had similar expression levels as ChaC2 (Figure 7E). Taken together, the ChaC2 mutant’s data suggest that ChaC2 promotes breast cancer cell proliferation, while its active site mutants reduce this effect. As such, the GSH degradation activity of ChaC2 was shown to regulate the growth of MCF-7 breast cancer cell lines. This will be discussed later in the discussion

### 3.8. Proposed Mechanism of Substrate Recognition and GSH Degradation of ChaC2

Although the conserved Glu83 residue is located far away from the interface of flexible loop2 in the crystallographic ChaC2 dimer, the ChaC2 E83Q mutation induced a conformational change; however, it did not significantly reduce the enzymatic activity, compared with the ChaC2 E74Q mutation. Considering that the ChaC2 E74Q and ChaC2 E83Q structures adopted a helical conformation in the flexible loop2 region similar to the structure of GGACT in complex with 5-l-oxoproline [12] (Figure 3 and Figure 5A), Glu83 is likely to play an important role in flexible loop2 dynamics and serve as an alternative catalytic residue. Furthermore, domain-swapped proteins are often considered as an open conformation, and, upon substrate- or ligand-binding, elicits conformational changes, resulting in a closed conformation [49]. Therefore, there is a good possibility that ChaC2 would switch its conformational status at the early stage of substrate GSH binding with the aid of the flexible loop region to bring the activity site residues in close proximity to GSH. The ChaC2 E74Q and ChaC2 E83Q structures would be reminiscent of a closed conformation.

**Figure 8:**
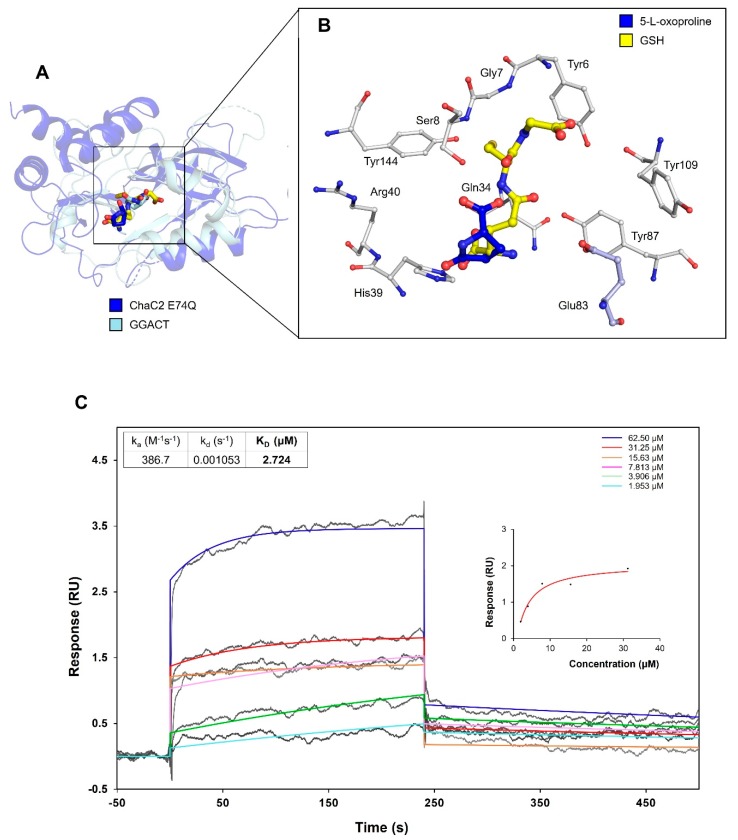
Docking model of ChaC2 E74Q with GSH and 5-l-oxoproline. (**A**) The docking models of GSH-ChaC2 E74Q and 5-l-oxoproline-ChaC2 E74Q have been superimposed onto the structure of GGACT in complex with 5-l-oxoproline. ChaC2 E74Q and GGACT are colored in blue and pale cyan, respectively. The GSH (docked into ChaC2 E74Q), 5-l-oxoproline (docked into ChaC2 E74Q), and 5-l-oxoproline (bound to GGACT) are denoted as yellow, blue, and pale cyan stick models, respectively. The docking binding energies of GSH and 5-l-oxoproline with ChaC2 are −6.3 kcal/mol and −5.2 kcal/mol, respectively. (**B**) Close-up view of the active site cavity of ChaC2. The ChaC2 E74Q residues recognizing GSH/5-l-oxoproline and catalytic Glu83 are shown as stick models with white and light blue colors, respectively. (**C**) SPR experiments with ChaC2 E74Q E83Q and GSH. Various concentrations of free GSH were applied to immobilized ChaC2. Sensorgrams for 1.953, 3.906, 7.813, 15.63, 31.25, and 62.50 μM GSH are shown in different colors. The K_D_ value of GSH to ChaC2 E74Q E83Q was calculated as 2.724 μM.

To further elucidate the catalytic mechanism and the interaction mode between ChaC2 and GSH or 5-l-oxoproline (a product of the GSH degradation reaction by ChaC enzyme [15]), we generated ChaC2 E74Q and ChaC2 E83Q mutants in order to obtain their substrate or product complex. We attempted to determine the structure of the ChaC2 E74Q or ChaC2 E83Q mutant in complexes with GSH or 5-l-oxoproline, but failed. As an alternative, we conducted simulated docking experiments and SPR experiments. We used the ChaC2 E74Q structure for the docking study, since the ChaC2 E74Q mutant still retains the GSH degradation activity of ChaC2, and the Glu83 residue in the ChaC2 E74Q mutant structure may act as an alternative active site, as in the aforementioned observations. To validate the docking program *Autodock Vina*, we implemented a control docking experiment with 5-l-oxorproline in the GGACT complex structure (PDB ID: 3JUC). 5-l-oxorproline was predicted to dock into the active site of GGACT as in the actual complex structure with a binding energy of −5.1 kcal/mol (Figure S7). The docking results predicted that GSH or 5-l-oxorproline would bind to ChaC2 in very similar mode to that of the GGACT-5-l-oxorproline complex structure (Figure 8A). In the docking model, GSH or 5-l-oxorproline are recognized/stabilized by conserved active site residues Tyr6, Gly7, Ser8, Glu34, His39, Arg40, Tyr87, Tyr109, and Tyr144 of ChaC2, which also played similar roles in the GGACT-5-l-oxoproline complex structure (Figure 5B and Figure 8B). In addition, the binding energy of GSH (−6.3 kcal/mol) was lower than that of 5-l-oxoproline (−5.2 kcal/mol), which suggests that GSH exhibits a higher binding affinity to ChaC2 than 5-l-oxoproline. We next examined the binding affinity of GSH and 5-l-oxoproline with ChaC2 by SPR. The SPR results showed that there is an interaction between GSH and ChaC2. Meanwhile, the interaction of 5-l-oxoproline with ChaC2 seemed too weak to be analyzed by SPR. Interestingly, ChaC2, ChaC2 E74Q, and ChaC2 E83Q showed a low binding response to GSH, whereas the ChaC2 E74Q E83Q showed a significantly high binding affinity with the calculated K_D_ value of 2.7 μM (Figure 8C). The docking and SPR results imply that ChaC2 binds to GSH and the binding is instantaneous, and the release of product 5-l-oxoproline during the GSH degradation reaction is rapid. Furthermore, the mutation of active-site residues strongly bind to GSH and would inhibit the GSH degradation activity, resulting in higher binding affinity.

## 4. Discussion

### 4.1. Role of the Flexible Loop2 Region of ChaC2 and Its Effects on Catalysis

The overall structure of ChaC2 and its active site cavity are very similar to previously determined structures of GGCT enzymes. However, ChaC2 adopted a unique quaternary structure wherein two ChaC2 monomers exchanged their loop1 and loop2 structures, resulting in a more flexible and open active site cavity. It has been previously reported that the exchanging loop of the HRAS-like tumor suppressor 3 (HRASLS) enzyme structure would be flexible or disordered without a bound ligand, and is involved in the re-arrangements to shield the active site from water molecules during the catalytic reaction steps [50]. According to the earlier structural study of yeast ChaC2, a similar short loop was also observed and suggested to change its conformation upon ligand binding, contributing to enhanced enzyme specificity [13]. To our surprise, the ChaC2 E74Q and ChaC2 E83Q mutants not only caused structural rearrangements but also had an effect on the enzymatic function in vitro and in vivo, compared to the ChaC2 wild type. However, the ChaC2 E74Q and ChaC2 E83Q mutants did not abolish the GSH degradation activity, while the equivalent Glu residue mutations of ChaC1 and GGCT resulted in the complete loss of this activity [11,15]. Collectively, our ChaC2 structures provide valuable insight into the mechanism by which the flexibility of the long extended loop2 regulates catalytic activities and maintains a constant GSH degradation rate. To initiate the catalysis, ChaC2 needs to undergo a conformational change in the flexible loop region, which allows and aids the synchronous movement of the substrate inside and outside of the active sites. On the other hand, the long flexible loop2 may also block the binding of substrates to ChaC2 and thereby limit the GSH degradation capacity of ChaC2. Therefore, the movement caused by the flexible loop would be closely accompanied by the steady and constant GSH degradation activity of ChaC2 in GSH metabolism.

The ChaC family belongs to the GGCT family but exclusively mediates the GSH degradation [13,15]. Although the catalytic cavity of ChaC2 is generally conserved among other GGCT enzymes, a long flexible loop2 has not been observed in the cavity of any GGCT enzyme. When the oligomeric states of ChaC1 and ChaC2 were compared using size-exclusion chromatography, ChaC2 mainly existed as a monomer in solution, while ChaC1 existed not only as a dimer but also as a tetramer (Figure S6). In addition, ChaC2 is constitutively expressed and exhibits lower activity in the “housekeeping” GSH metabolism, while ChaC1 is only expressed in response to specific stresses or in tumors. These observations reinforce the notion that dimerization may be an important determinant in the regulation of ChaC enzyme activity and substrate specificity. ChaC enzymes form dimers or higher oligomers for enzymatic functions in stressful or tumor conditions for enzyme crowding. The lower activity of ChaC2 compared to ChaC1 may be partly caused by the flexible loop2, which further results in dimer/monomer equilibrium and open/closed conformation of the active site according to the oligomeric status.

Our structural analyses of ChaC2 with long flexible loop2 revealed a novel structural feature of the GGCT protein, which provides valuable insights regarding the contribution of flexible loop rearrangements for modulating enzyme activity. In addition, the long flexible loop2 conformation provides the first structural snapshot of the dynamic flexibility during the GSH degradation reaction mediated by ChaC2. We believe that the dynamics of the ChaC2 flexible loop2 plays a specific and efficient role in GSH degradation. To further validate the importance of the oligomeric status of ChaC2 in GSH degradation, the simulation of the molecular dynamics of the monomer/dimer transition in the presence or absence of GSH would be helpful.

### 4.2. Correlation of ChaC2, GSH Degradation, and Breast Cancer

ChaC family proteins have been considered attractive targets for cancer therapies due to their influence on cancer cell proliferation [18,21,47]. Overexpression of ChaC1 increased the proliferation of breast cancer and ovarian cancer cell lines; however, it is not yet fully understood why the overexpression of the ChaC family proteins promotes the proliferation of certain cancer cell lines [18]. Interestingly, the clinical database demonstrate that the ChaC2 level is significantly high in invasive ductal breast carcinoma. In addition, the ChaC2 level in breast cancer tissue was markedly associated with breast cancer survival. From cellular experiments, we found that the overexpression of ChaC2 in the MCF-7 breast cancer cell line (an invasive ductal carcinoma) induced cell proliferation. However, the overexpression of ChaC2 harboring mutations at the active site did not induce cell proliferation, unlike the wild type ChaC2. These results indicate that ChaC2 modulates the growth of breast cancer cells via its GSH degradation activity in the GSH metabolism. ChaC2 knockdown has been shown to downregulate Nrf2, a master of the antioxidant response [10]. In breast cancer cells, Nrf2 has been also shown to be overexpressed and promote cell proliferation [51,52]. Overexpression of ChaC2 mutants at the active site would result in the increase of the intracellular GSH level and the inhibition of Nrf2, which would elicit anti-proliferative effects on cancer cells. In summary, our data firstly revealed that overexpression of ChaC2 unfavorably influences the survival of breast cancer patients due to its capacity of promoting cancer cell proliferation and modulating the antioxidant system. Our findings provide beneficial insights into developing new treatment strategies for breast cancer patients by inhibiting the glutathione degradation activity of ChaC family enzymes.

## 5. Conclusions

Here, we characterized human ChaC2, a GGCT enzyme with a unique structure. We determined the crystal structure of ChaC2 with different conformations in the flexible loop2 region. Based on the structural analyses and biochemical assay results, we elucidated key residues of ChaC2 for its glutathione-degradation activity. In addition, we found a close relationship between ChaC2 function and breast cancer from our cell proliferation assay results and previously published breast cancer databases. Collectively, our researches shed light on the molecular basis of a glutathione-controlling ChaC2 enzyme and suggest its role in cancer cell proliferation.

## Figures and Tables

**Table 1:** Multi-Wavelength anomalous diffraction phasing statistics.

	SeMet-λ1 (Peak)	SeMet-λ2 (Edge)	SeMet-λ3 (Remote)
Data Collection			
Wavelength (Å)	0.9793	0.9795	0.9717
Space Group	*P*3_1_21		
Cell dimensions			
a, b, c (Å)	64.12, 64.12, 103.78 90.00, 90.00, 120.00		
α, ß, γ (°)			
Resolution (Å) *	50.00–2.80 (2.85–2.80)	50.00–2.95 (3.00–2.95)	50.00–3.00 (3.05–3.00)
Rsym *	0.130 (0.948)	0.130 (0.854)	0.148 (0.145)
Total no. observations	136,727	117,519	111,444
Total no. unique reflections	11,761	10,110	9597
*I/σ(I)* *	14.7 (3.1)	14.7 (3.1)	47.7 (3.5)
Completeness (%) *	99.9 (100.0)	99.9 (100.0)	99.9 (100.0)
Multiplicity *	21.2 (22.3)	21.1 (22.0)	21.0 (21.9)
CC_1/2_	0.997 (0.990)	0.996 (0.985)	0.996 (0.980)

* Values in parentheses correspond to highest-resolution shell. X-ray diffraction experiment was performed at PAL–7A, Pohang Photon Factory (Pohang, Korea).

**Table 2:** Data collection and refinement statistics of ChaC2 structures.

Structures	ChaC2 (PDB ID: 6K95)	ChaC2 E74Q (PDB ID: 6KYO)	ChaC2 E83Q (PDB ID: 6KY1)
Data Collection			
Beam line	Spring–8	PAL–7A	PAL–5C
Wavelength (Å)	0.9000	0.9793	0.9795
Space Group	*C*2	*P*3_1_	*P*3_1_
Cell dimensions			
a, b, c (Å)	108.84, 62.16, 103.62	72.92, 72.92,104.25	72.64, 72.64, 104.13
α, ß, γ (°)	90.00, 90.01, 90.00	90.00, 90.00, 120.00	90.00, 90.00, 120.00
Resolution (Å) *	50.00–2.30 (2.34–2.30)	50.00–2.06 (2.10–2.06)	50.00–2.04 (2.08–2.04)
R_sym_ *	0.056 (0.403)	0.079 (0.826)	0.070 (0.563)
Total no. reflections	164,479	363,215	241,362
Total no. unique reflections	31,202	38,639	38,900
I/σ(I) *	23.0 (5.2)	22.7 (8.1)	42.5 (4.2)
Completeness (%) *	99.5 (99.9)	99.7 (99.8)	99.9 (100.0)
Multiplicity *	5.3 (5.2)	9.4 (9.4)	6.2 (6.0)
CC_1/2_	0.995 (0.993)	0.996 (0.95)	0.99 (0.96)
**Refinement**			
Resolution range (Å)	37.41–2.30	31.59–2.06	31.48–2.04
R_work_/R_free_^†^	0.239/0.268	0.224/0.258	0.225/0.255
No. atoms			
Protein	4177	4144	4188
Water	16	77	85
Average B factors (Å^2^)	47.36	36.84	35.56
Protein	47.30	36.6	35.07
Water	69.26	51.00	59.08
R.m.s. deviations			
Bond length (Å)	0.011	0.008	0.009
Bond angles (°)	1.440	1.184	1.300
Ramachandran Favored/outlier	97.45/0.0	98.22/0.0	96.8/0.0

* Values in parentheses are for the highest resolution shell. ^†^ About 5% of the reflections were excluded from the refinement for *R*_free_ calculation.

## Supplementary Materials

The following are available online at https://www.mdpi.com/2218-273X/10/1/31/s1, Figure S1: Secondary structure and solvent accessibility predictions using the *PSIPRED* and *SABLE* server, Figure S2: The close-view of long flexible loop2, Figure S3: 2*mFo-DFc* electron densities of ChaC2 E74Q (left) and ChaC2 E83Q (right) contoured at 2.0*σ*, Figure S4: Sequence alignment of human ChaC2 (UniProt ID: Q8WUX2) and four representative GGCT proteins: human GGCT (UniProt ID: 075223), human GGACT (UniProt ID: Q9BVM4), yeast GCG1 (UniProt ID: P32656), and human ChaC1 (UniProt ID: Q9BUX1), Figure S5: Overexpression of ChaC2 in ChaC2-transiently transfected HEK293 cell lines, Figure S6: The oligomeric status of human ChaC1 and ChaC2, Figure S7: The control docking experiment result of the human GGACT with 5-l-oxoproline complex, Figure S8: SPR experiments with ChaC2 and GSH.

## References

[1] Liu Y., Hyde A.S., Simpson M.A., Barycki J.J., Townsend D.M., Tew K.D. (2014). Chapter Two—Emerging Regulatory Paradigms in Glutathione Metabolism. Advances in Cancer Research.

[2] Messina J.P., Lawrence D.A. (1989). Cell cycle progression of glutathione-depleted human peripheral blood mononuclear cells is inhibited at S phase. J. Immunol..

[3] Townsend D.M., Tew K.D., Tapiero H. (2003). The importance of glutathione in human disease. Biomed. Pharmacother..

[4] Bansal A., Simon M.C. (2018). Glutathione metabolism in cancer progression and treatment resistance. J. Cell Biol..

[5] Traverso N., Ricciarelli R., Nitti M., Marengo B., Furfaro A.L., Pronzato M.A., Marinari U.M., Domenicotti C. (2013). Role of glutathione in cancer progression and chemoresistance. Oxidative Med. Cell. Longev..

[6] Kumar B.A., Amandeep K. (2017). Glutathione Degradation. Antioxid. Redox Signal..

[7] Martin M.N., Saladores P.H., Lambert E., Hudson A.O., Leustek T. (2007). Localization of Members of the γ-Glutamyl Transpeptidase Family Identifies Sites of Glutathione and Glutathione *S*-Conjugate Hydrolysis. Plant. Physiol..

[8] Han B., Luo G., Zheng-Zheng S., Barrios R. (2002). Gamma-glutamyl leukotrienase, a novel endothelial membrane protein, is specifically responsible for leukotriene D(4) formation in vivo. Am. J. Pathol..

[9] Wickham S., West M.B., Cook P.F., Hanigan M.H. (2011). Gamma-glutamyl compounds: Substrate specificity of gamma-glutamyl transpeptidase enzymes. Anal. Biochem..

[10] Wang C.-K., Yang S.-C., Hsu S.-C., Chang F.-P., Lin Y.-T., Chen S.-F., Cheng C.-L., Hsiao M., Lu F.L., Lu J. (2017). CHAC2 is essential for self-renewal and glutathione maintenance in human embryonic stem cells. Free Radic. Biol. Med..

[11] Oakley A.J., Yamada T., Liu D., Coggan M., Clark A.G., Board P.G. (2008). The Identification and Structural Characterization of C7orf24 as γ-Glutamyl Cyclotransferase: An Essential Enzyme in the γ-Glutamyl Cycle. J. Biol. Chem..

[12] Oakley A.J., Coggan M., Board P.G. (2010). Identification and Characterization of γ-Glutamylamine Cyclotransferase, an Enzyme Responsible for γ-Glutamyl-ϵ-lysine Catabolism. J. Biol. Chem..

[13] Kaur A., Gautam R., Srivastava R., Chandel A., Kumar A., Karthikeyan S., Bachhawat A.K. (2017). ChaC2, an Enzyme for Slow Turnover of Cytosolic Glutathione. J. Biol. Chem..

[14] Chi Z., Byrne S.T., Dolinko A., Harraz M.M., Kim M.-S., Umanah G., Zhong J., Chen R., Zhang J., Xu J. (2014). Botch Is a γ-Glutamyl Cyclotransferase that Deglycinates and Antagonizes Notch. Cell Rep..

[15] Kumar A., Tikoo S., Maity S., Sengupta S., Sengupta S., Kaur A., Kumar Bachhawat A. (2012). Mammalian proapoptotic factor ChaC1 and its homologues function as γ-glutamyl cyclotransferases acting specifically on glutathione. EMBO Rep..

[16] Joo N.E., Ritchie K., Kamarajan P., Miao D., Kapila Y.L. (2012). Nisin, an apoptogenic bacteriocin and food preservative, attenuates HNSCC tumorigenesis via CHAC1. Cancer Med..

[17] Mungrue I.N., Pagnon J., Kohannim O., Gargalovic P.S., Lusis A.J. (2009). CHAC1/MGC4504 Is a Novel Proapoptotic Component of the Unfolded Protein Response, Downstream of the ATF4-ATF3-CHOP Cascade. J. Immunol..

[18] Goebel G., Berger R., Strasak A.M., Egle D., Müller-Holzner E., Schmidt S., Rainer J., Presul E., Parson W., Lang S. (2012). Elevated mRNA expression of CHAC1 splicing variants is associated with poor outcome for breast and ovarian cancer patients. Br. J. Cancer.

[19] Crawford R.R., Prescott E.T., Sylvester C.F., Higdon A.N., Shan J., Kilberg M.S., Mungrue I.N. (2015). Human CHAC1 Protein Degrades Glutathione, and mRNA Induction Is Regulated by the Transcription Factors ATF4 and ATF3 and a Bipartite ATF/CRE Regulatory Element. J. Biol. Chem..

[20] Nomura Y., Hirata Y., Kiuchi K., Oh-hashi K. (2016). Translational and post-translational regulation of mouse cation transport regulator homolog 1. Sci. Rep..

[21] Liu S., Fei W., Shi Q., Li Q., Kuang Y., Wang C., He C., Hu X. (2017). CHAC2, downregulated in gastric and colorectal cancers, acted as a tumor suppressor inducing apoptosis and autophagy through unfolded protein response. Cell Death Dis..

[22] L’Espérance S., Bachvarova M., Tetu B., Mes-Masson A.-M., Bachvarov D. (2008). Global gene expression analysis of early response to chemotherapy treatment in ovarian cancer spheroids. BMC Genom..

[23] Bailey H.H., Ripple G., Tutsch K.D., Arzoomanian R.Z., Alberti D., Feierabend C., Mahvi D., Schink J., Pomplun M., Mulcahy R.T. (1997). Phase I Study of Continuous-Infusion *L*-*S*,*R*-Buthionine Sulfoximine With Intravenous Melphalan. JNCI J. Natl. Cancer Inst..

[24] The UniProt Consortium (2017). UniProt: the universal protein knowledgebase. Nucleic Acids Res..

[25] Sievers F., Wilm A., Dineen D., Gibson T.J., Karplus K., Li W., Lopez R., McWilliam H., Remmert M., Söding J. (2011). Fast, scalable generation of high-quality protein multiple sequence alignments using Clustal Omega. Mol. Syst. Biol..

[26] Gouet P., Courcelle E., Stuart D., Métoz F. (1999). ESPript: analysis of multiple sequence alignments in PostScript. Bioinformatics.

[27] Walden H. (2010). Selenium incorporation using recombinant techniques. Acta Crystallogr. Sect. D Biol. Crystallogr..

[28] McGuffin L.J., Bryson K., Jones D.T. (2000). The PSIPRED protein structure prediction server. Bioinformatics.

[29] Rafał A., Aleksey P., Jarosław M. (2005). Combining prediction of secondary structure and solvent accessibility in proteins. Proteins Struct. Funct. Bioinform..

[30] Rhodes D.R., Kalyana-Sundaram S., Mahavisno V., Varambally R., Yu J., Briggs B.B., Barrette T.R., Anstet M.J., Kincead-Beal C., Kulkarni P. (2007). Oncomine 3.0: genes, pathways, and networks in a collection of 18,000 cancer gene expression profiles. Neoplasia.

[31] Györffy B., Lanczky A., Eklund A.C., Denkert C., Budczies J., Li Q., Szallasi Z. (2010). An online survival analysis tool to rapidly assess the effect of 22,277 genes on breast cancer prognosis using microarray data of 1809 patients. Breast Cancer Res. Treat..

[32] Otwinowski Z., Minor W. (1997). [20] Processing of X-ray diffraction data collected in oscillation mode. Methods in Enzymology.

[33] Adams P.D., Afonine P.V., Bunkoczi G., Chen V.B., Davis I.W., Echols N., Headd J.J., Hung L.-W., Kapral G.J., Grosse-Kunstleve R.W. (2010). PHENIX: a comprehensive Python-based system for macromolecular structure solution. Acta Crystallogr. Sect..

[34] McCoy A.J., Grosse-Kunstleve R.W., Adams P.D., Winn M.D., Storoni L.C., Read R.J. (2007). Phaser crystallographic software. J. Appl. Crystallogr..

[35] Emsley P., Lohkamp B., Scott W.G., Cowtan K. (2010). Features and development of Coot. Acta Crystallogr. Sect. Biol. Crystallogr..

[36] Winn M.D., Ballard C.C., Cowtan K.D., Dodson E.J., Emsley P., Evans P.R., Keegan R.M., Krissinel E.B., Leslie A.G.W., McCoy A. (2011). Overview of the CCP4 suite and current developments. Acta Crystallogr. Sect. Biol. Crystallogr..

[37] Williams C.J., Headd J.J., Moriarty N.W., Prisant M.G., Videau L.L., Deis L.N., Verma V., Keedy D.A., Hintze B.J., Chen V.B. (2018). MolProbity: More and better reference data for improved all-atom structure validation. Protein Sci..

[38] Joosten R.P., Salzemann J., Bloch V., Stockinger H., Berglund A.-C., Blanchet C., Bongcam-Rudloff E., Combet C., Da Costa A.L., Deleage G. (2009). PDB_REDO: automated re-refinement of X-ray structure models in the PDB. J. Appl. Crystallogr..

[39] Rahman I., Kode A., Biswas S.K. (2007). Assay for quantitative determination of glutathione and glutathione disulfide levels using enzymatic recycling method. Nat. Protoc..

[40] Trott O., Olson A.J. (2010). AutoDock Vina: improving the speed and accuracy of docking with a new scoring function, efficient optimization and multithreading. J. Comput. Chem..

[41] Wu H.-Y., Liu M.-S., Lin T.-P., Cheng Y.-S. (2011). Structural and Functional Assays of AtTLP18.3 Identify Its Novel Acid Phosphatase Activity in Thylakoid Lumen. Plant Physiol..

[42] Krissinel E., Henrick K. (2005). Detection of Protein Assemblies in Crystals.

[43] Holm L., Rosenström P. (2010). Dali server: conservation mapping in 3D. Nucleic Acids Res..

[44] Ran R., Liu Y., Gao H., Kuang Q., Zhang Q., Tang J., Fu H., Zhang Z., He Q. (2015). PEGylated Hyaluronic Acid-Modified Liposomal Delivery System with Anti-γ-Glutamylcyclotransferase siRNA for Drug-Resistant MCF-7 Breast Cancer Therapy. J. Pharm. Sci..

[45] Matsumura K., Nakata S., Taniguchi K., Ii H., Ashihara E., Kageyama S., Kawauchi A., Yoshiki T. (2016). Depletion of γ-glutamylcyclotransferase inhibits breast cancer cell growth via cellular senescence induction mediated by CDK inhibitor upregulation. BMC Cancer.

[46] Hiromi I., Taku Y., Susumu N., Keiko T., Koushi H., Shugo T., Masayoshi M., Yuji N., Yuko T., Kosei I. (2018). A Novel Prodrug of a γ-Glutamylcyclotransferase Inhibitor Suppresses Cancer Cell Proliferation in vitro and Inhibits Tumor Growth in a Xenograft Mouse Model of Prostate Cancer. Chem. Med. Chem..

[47] Jahn B., Arvandi M., Rochau U., Fiegl H., Goebel G., Marth C., Siebert U. (2017). Development of a novel prognostic score for breast cancer patients using mRNA expression of CHAC1. J. Comp. Eff. Res..

[48] Chen M.-S., Wang S.-F., Hsu C.-Y., Yin P.-H., Yeh T.-S., Lee H.-C., Tseng L.-M. (2017). CHAC1 degradation of glutathione enhances cystine-starvation-induced necroptosis and ferroptosis in human triple negative breast cancer cells via the GCN2-eIF2α-ATF4 pathway. Oncotarget.

[49] Taylor C.A.t., Juang Y.-C., Earnest S., Sengupta S., Goldsmith E.J., Cobb M.H. (2015). Domain-Swapping Switch Point in Ste20 Protein Kinase SPAK. Biochemistry.

[50] Golczak M., Sears A.E., Kiser P.D., Palczewski K. (2015). LRAT-specific domain facilitates vitamin A metabolism by domain swapping in HRASLS3. Nat. Chem. Biol..

[51] Zhang C., Wang H.-J., Bao Q.-C., Wang L., Guo T.-K., Chen W.-L., Xu L.-L., Zhou H.-S., Bian J.-L., Yang Y.-R. (2016). NRF2 promotes breast cancer cell proliferation and metastasis by increasing RhoA/ROCK pathway signal transduction. Oncotarget.

[52] Zhang H.-S., Du G.-Y., Zhang Z.-G., Zhou Z., Sun H.-L., Yu X.-Y., Shi Y.-T., Xiong D.-N., Li H., Huang Y.-H. (2018). NRF2 facilitates breast cancer cell growth via HIF1ɑ-mediated metabolic reprogramming. Int. J. Biochem. Cell Biol..

