# Supplementary material for: Structural and Functional Analyses of Human ChaC2 in Glutathione Metabolism
Source: Biomolecules. 2019 Dec 24;10(1):31. doi: 10.3390/biom10010031 (PMC7022552; doi:10.3390/biom10010031)
Supplement: Supplementary file 1 [file biomolecules-10-00031-s001.pdf]

Supplementary Materials:

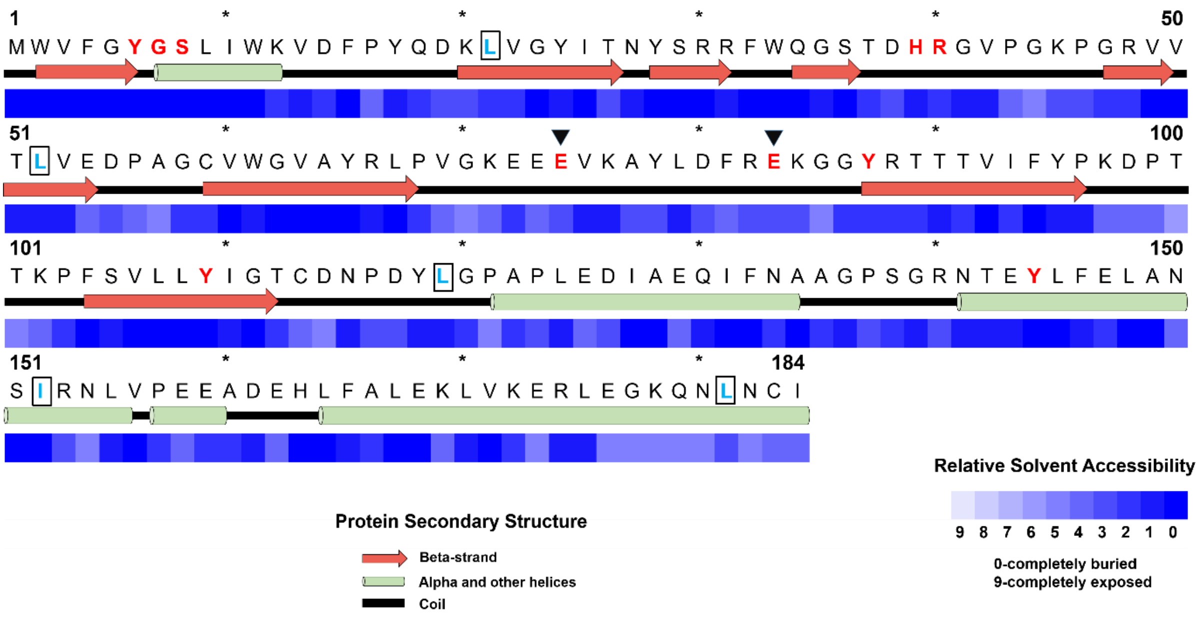

Figure S1. Secondary structure and solvent accessibility predictions using the *PSIPRED* and *SABLE* server. The predicted helices, β-strands, and coils are shown in pale green, deep salmon, black, respectively, below the ChaC2 amino acid sequence. The residues predicted to be buried or exposed are denoted in a gradient of white to blue colors. The conserved key residues among the GGCT proteins are shown in red (see also Fig. S4). Glu83 and Glu74 are indicated with black triangles. The residues substituted with Met in our study are colored in cyan and shown in black squares.

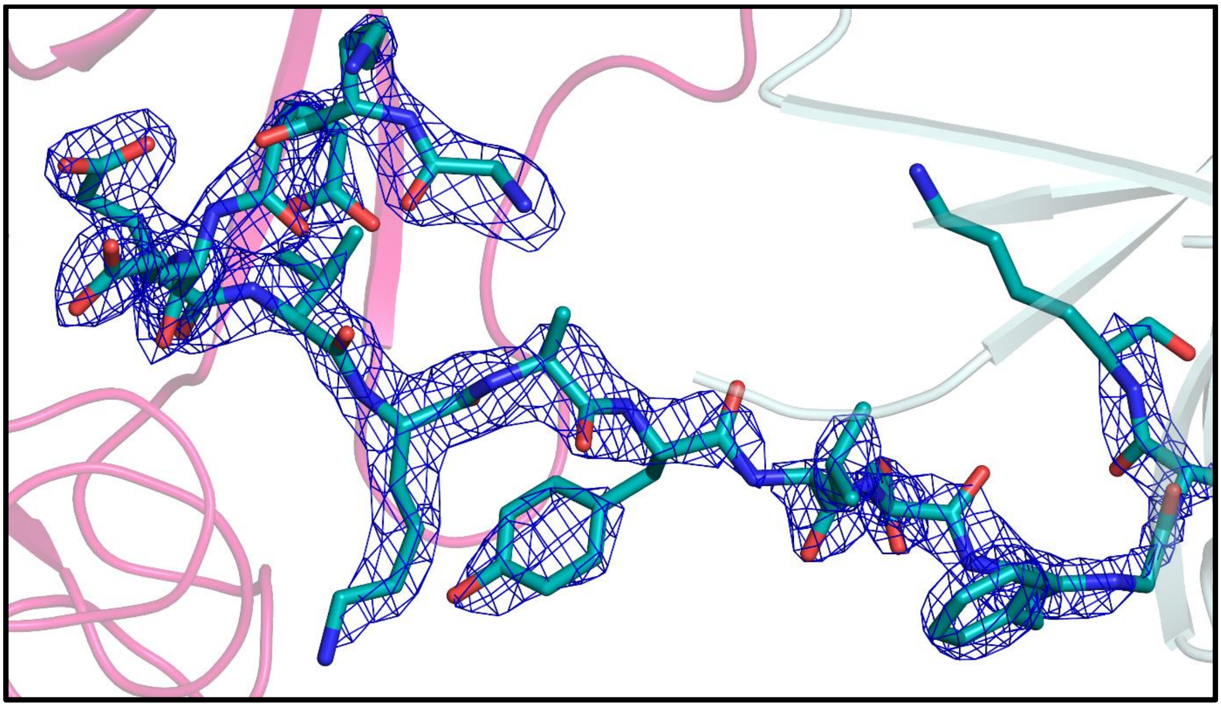

Figure S2. The close-view of long flexible loop2. 2*mFo-DFc* electron densities of flexible loop were contoured at *2.0σ*.

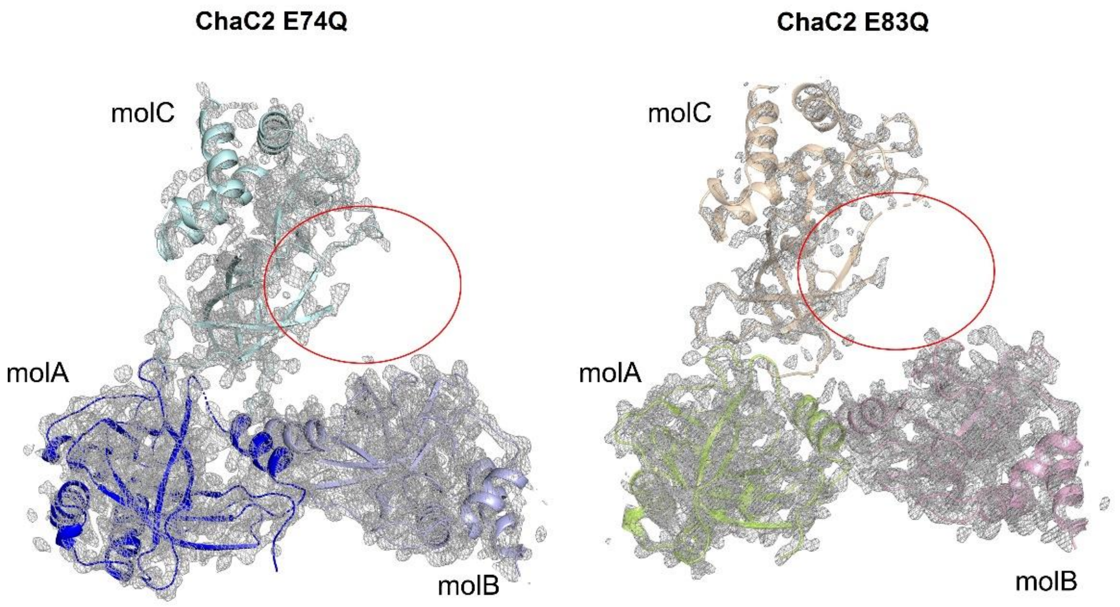

Figure S3. 2*mFo-DFc* electron densities of ChaC2 E74Q (left) and ChaC2 E83Q (right) contoured at *2.0σ*. The loop2 regions of ChaC2 E74Q and ChaC2 E83Q are indicated by red circles.

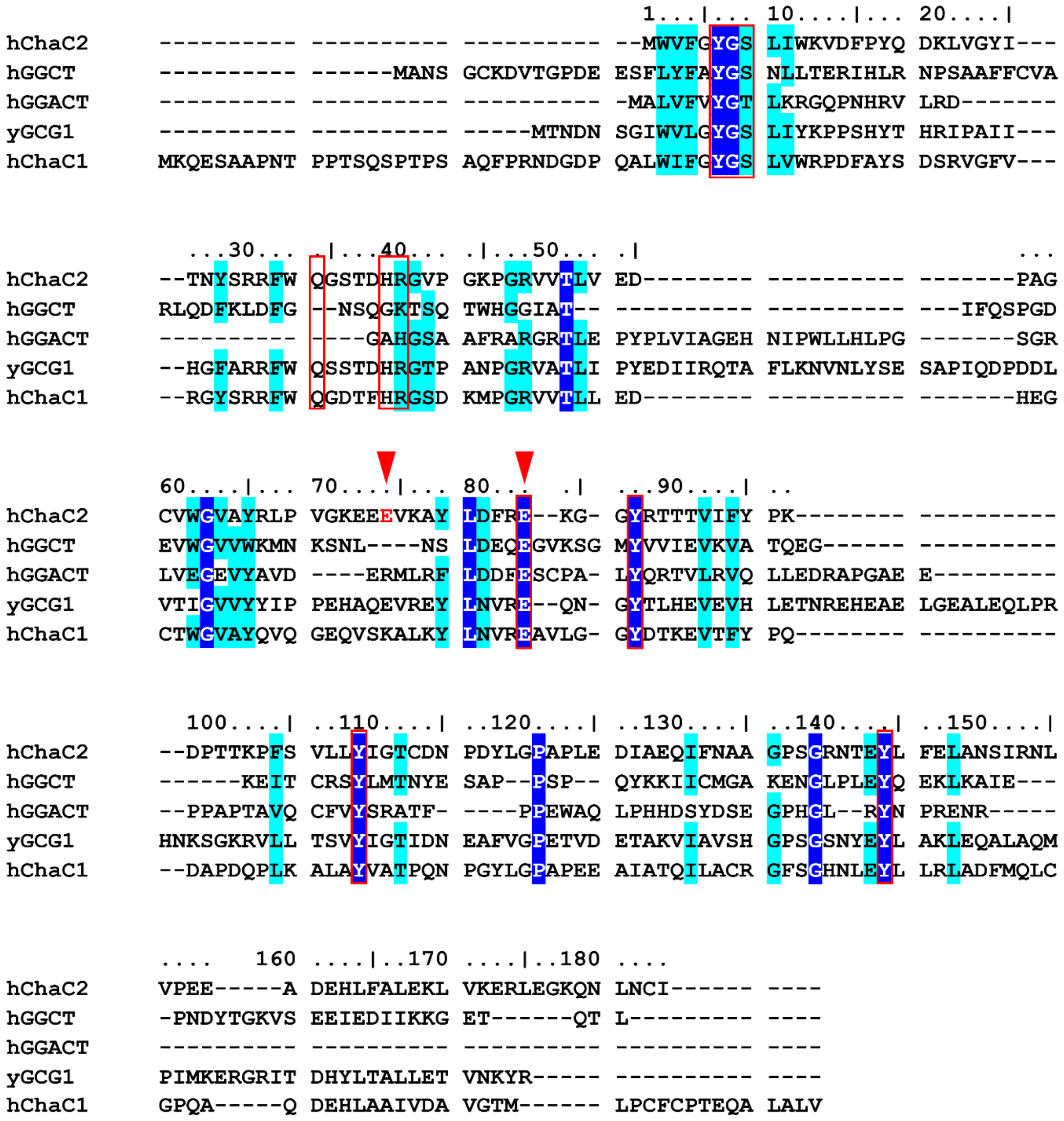

Figure S4. Sequence alignment of human ChaC2 (UniProt ID: Q8WUX2) and four representative GGCT proteins: human GGCT (UniProt ID: 075223), human GGACT (UniProt ID: Q9BVM4), yeast GCG1 (UniProt ID: P32656), and human ChaC1 (UniProt ID: Q9BUX1). Among these five proteins, the identical/similar residues are shaded in blue/cyan, respectively. The residues involved in GSH binding are additionally boxed in red square. The Glu74 and Glu83 of human ChaC2 are indicated with red triangles. The alignment was performed by *Clustal Omega* software with *ESPRIPT3*.

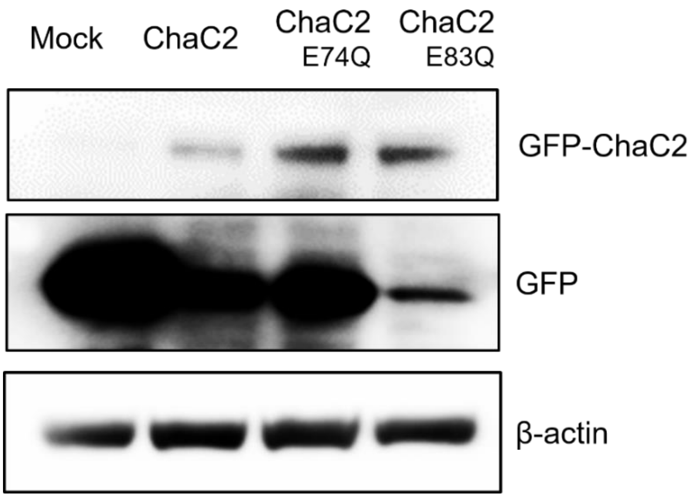

Figure S5. Overexpression of ChaC2 in ChaC2-transiently transfected HEK293 cell lines. The cell lysates of the Mock, ChaC2, ChaC2 E74Q, and ChaC2 E83Q-transfected cells were analyzed by western blotting using a GFP antibody to detect GFP-tagged ChaC2 proteins.

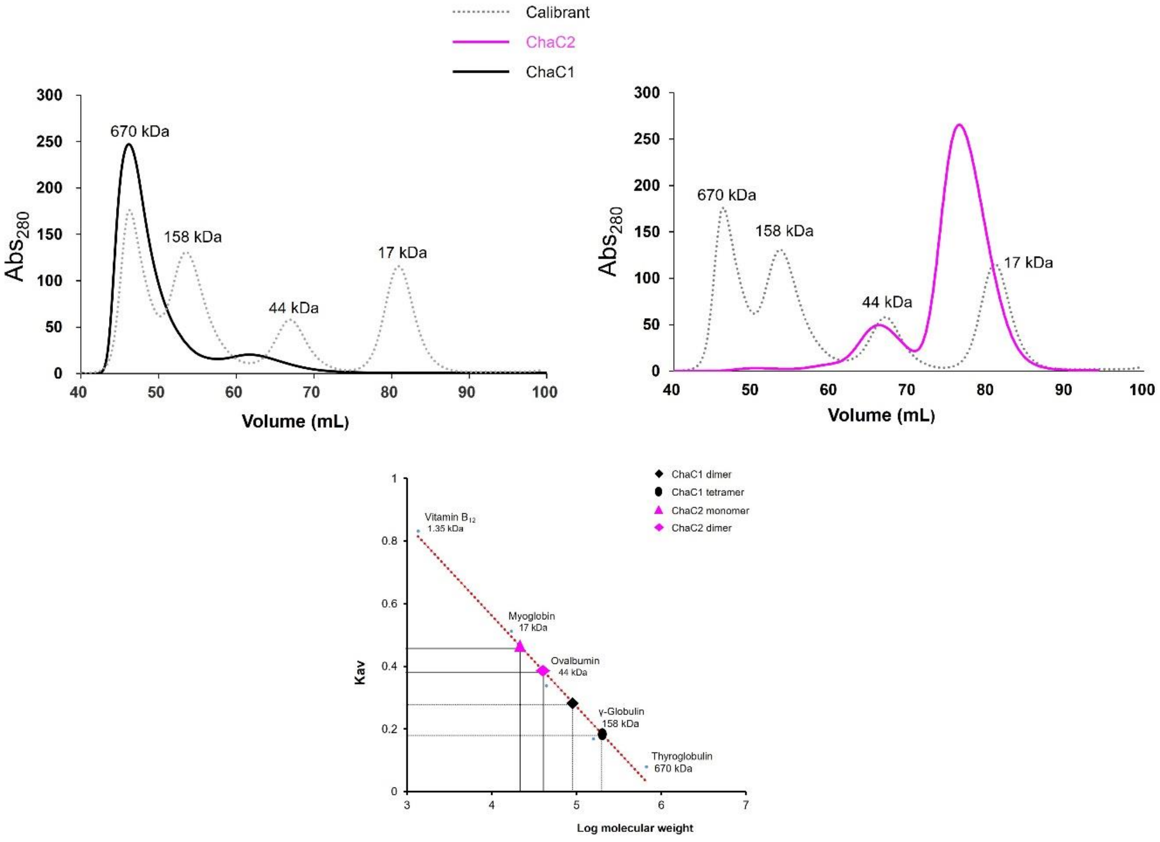

Figure S6. The oligomeric status of human ChaC1 (26 kDa) and ChaC2 (20 kDa). The size-exclusion chromatograms of ChaC1 (left) and ChaC2 (right) are shown. ChaC1 and ChaC2 were loaded onto a HiLoad 16/600 Superdex 75 pg column at a flow rate of 1 mL/min. The eluted proteins were monitored at 280 nm. The chromatograms of ChaC1 and ChaC2 are indicated in black and magenta, respectively. The chromatogram of calibration mixture (thyroglobulin 670 kDa, γ-globulin 158 kDa, ovalbumin 44 kDa, and myoglobin 17 kDa) are shown in gray. The calibration/selectivity plot for standard proteins and ChaC2 proteins is shown in the panel below.

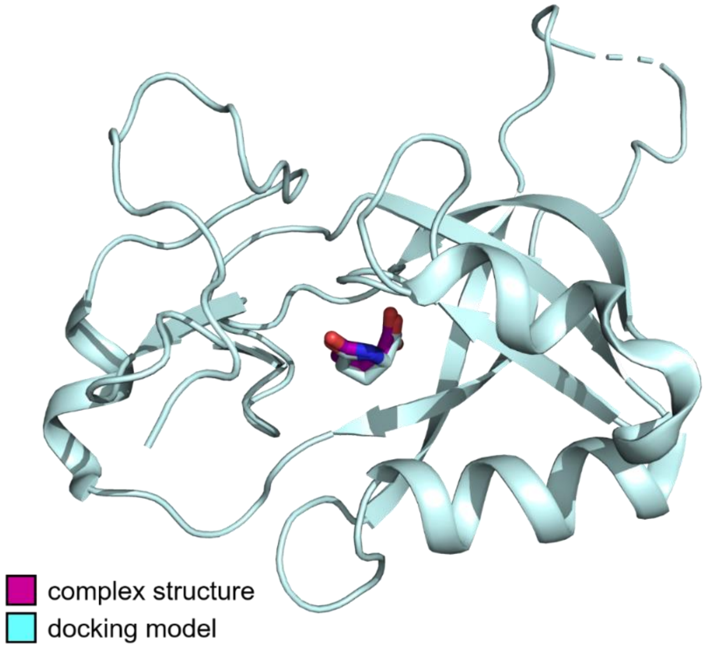

Figure S7. The control docking experiment result of the human GGACT with 5-L-oxoproline complex. The 5-L-oxoprolines in the GGACT complex structure and from our control docking experiment are shown by magenta and cyan stick models, respectively. Oxygen atoms are colored in red. The docking binding energy of 5-L-oxoproline with GGACT is – 5.1 kcal/mol.

Table S1. Interaction of crystallographic ChaC2 dimer that makes close contacts via long flexible loop2, calculated by the PISA web server.

Hydrogen Bond			Salt Bridge Interaction		
Monomer A	Monomer B	Distance (Å)	Monomer A	Monomer B	Distance (Å)
Ser8 [N]	Glu74 [OE2]	2.80	Lys12 [NZ]	Glu73 [OE2]	2.57
Ser8 [OG]	Glu74 [OE1]	3.06	Arg40 [NE]	Glu73 [OE2]	3.51
Ser8 [OG]	Glu73 [N]	2.90	Arg40 [NE]	Glu73 [OE1]	2.60
Ser8 [OG]	Lys71 [O]	3.56	Arg40 [NH2]	Glu73 [OE2]	2.73
Arg40 [NE]	Glu73 [OE1]	2.60	Arg40 [NH2]	Glu73 [OE1]	3.43
Arg40 [NH2]	Glu32 [OE2]	3.73	Glu73 [OE2]	Lys12 [NZ]	2.57
Lys71 [NZ]	Ser8 [OG]	3.56	Glu73 [OE1]	Arg40 [NE]	2.60
Glu73 [N]	Ser8 [OG]	2.90	Glu73 [OE2]	Arg40 [NE]	3.51
Tyr109 [OH]	Lys76 [O]	3.35	Glu73 [OE1]	Arg40 [NH2]	3.51
Tyr144 [OH]	Glu74 [OE2]	2.30	Glu73 [OE2]	Arg40 [NH2]	3.43
Lys71 [O]	Ser8 [OG]	3.61	Lys71 [NZ]	Asp14 [OD1]	3.47
Glu74 [OE1]	Ser8 [OG]	3.06	Lys71 [NZ]	Asp14 [OD2]	3.53
Glu74 [OE2]	Tyr144 [OH]	2.60	Lys76 [NZ]	Asp117 [OD1]	3.78
Glu73 [OE1]	Arg40 [NE]	2.60	Lys76 [NZ]	Asp117 [OD2]	2.72
Glu73 [OE2]	Arg40 [NH2]	2.73	Asp14 [OD2]	Lys71 [NZ]	3.53
Asp117 [OD2]	Lys76 [NZ]	2.72	Asp14 [OD1]	Lys71 [NZ]	3.47
Ser8 [OG]	Glu73 [N]	2.90	Asp117 [OD2]	Lys76 [NZ]	2.72
Lys76 [O]	Tyr109 [OH]	3.35	Asp117 [OD1]	Lys76 [NZ]	3.78
Lys12 [NZ]	Lys71 [O]	3.10			
Lys12 [NZ]	Glu73 [OE2]	2.57			
Lys76 [NZ]	Asp117 [OD2]	2.49			
Glu73 [N]	Lys12 [NZ]	2.57			

Table S2. Structural similarity of ChaC2 to other known structures in PDB using the *Dali* server

**Proteins**	**Z-score**	**Sequence identity (%)**	**PDB IDs**
Yeast glutathione-specific γ-glutamylcyclotransferase	19.2	38	5HWI
Human γ-glutamylcyclotransferase C7orf24	12.0	21	2PN7
Bacillus subtilis Ykqa protein	11.6	23	2QIK
Human hypothetical LOC79017 protein	11.4	21	2I5T
Arabidopsis thaliana AT5G39720.1 protein	7.7	17	2G0Q
Human γ-glutamylaminecyclotransferase	7.2	14	3JUC
Pyrococcus horikoshii uncharacterized PH0828 protein	6.9	16	1V30
Arabidopsis thaliana At3g28950.1 protein	6.6	19	2JQV
Kluyveromyces lactis allophanate hydrolase	6.1	9	4IST
Escherichia coli glutamyl-tRNA aminotransferase C4763	5.6	11	5C5Z
Escherichia coli hypothetical UPF0131 protein	5.4	23	1XHS

-. Structures with Z-scores over 5 were selected from the *Dali* results.
